# PINK1‐Dependent Mitophagy Regulates the Migration and Homing of Multiple Myeloma Cells via the MOB1B‐Mediated Hippo‐YAP/TAZ Pathway

**DOI:** 10.1002/advs.201900860

**Published:** 2020-01-23

**Authors:** Shengjun Fan, Trevor Price, Wei Huang, Michelle Plue, Jonathan Warren, Pasupathi Sundaramoorthy, Barry Paul, Daniel Feinberg, Nancie MacIver, Nelson Chao, Dorothy Sipkins, Yubin Kang

**Affiliations:** ^1^ Division of Hematologic Malignancies and Cellular Therapy Department of Medicine Duke University Medical Center Durham NC 27710 USA; ^2^ Shared Materials Instrumentation Facility Pratt School of Engineering Duke University Durham NC 27708 USA; ^3^ Department of Pediatrics Duke University Durham NC 27710 USA

**Keywords:** Hippo pathway, migration and homing, mitophagy, MOB1B, multiple myeloma, PINK1, YAP/TAZ

## Abstract

The roles of mitochondrial dysfunction in carcinogenesis remain largely unknown. The effects of PTEN‐induced putative kinase 1 (PINK1)‐dependent mitophagy on the pathogenesis of multiple myeloma (MM) are determined. The levels of the PINK1‐dependent mitophagy markers *PINK1* and parkin RBR E3 ubiquitin protein ligase (*PARK2)* in CD138^+^ plasma cells are reduced in patients with MM and correlate with clinical outcomes in myeloma patients. Moreover, the induction of PINK1‐dependent mitophagy with carbonylcyanide‐*m*‐chlorophenylhydrazone (CCCP) or salinomycin, or overexpression of PINK1 leads to inhibition of transwell migration, suppression of myeloma cell homing to calvarium, and decreased osteolytic bone lesions. Furthermore, genetic deletion of *pink1* accelerates myeloma development in a spontaneous X‐box binding protein‐1 spliced isoform (*XBP‐1s*) transgenic myeloma mouse model and in VK*MYC transplantable myeloma recipient mice. Additionally, treatment with salinomycin shows significant antimyeloma activities in vivo in murine myeloma xenograft models. Finally, the effects of PINK1‐dependent mitophagy on myeloma pathogenesis are driven by the activation of the Mps one binder kinase activator (MOB1B)‐mediated Hippo pathway and the subsequent downregulation of Yes‐associated protein (YAP)/transcriptional co‐activator with PDZ‐binding motif (TAZ) expression. These data provide direct evidence that PINK1‐dependent mitophagy plays a critical role in the pathogenesis of MM and is a potential therapeutic target.

## Graphical abstract

The important role of PTEN‐induced putative kinase 1 (PINK1)‐dependent mitophagy in regulating multiple myeloma progression and its effects on the Mps one binder kinase activator (MOB1B) Hippo‐Yes‐associated protein (YAP)/transcriptional co‐activator with PDZ‐binding motif (TAZ) pathway are summarized, with an emphasis on myeloma cell homing and migration. This study provides fundamental insights and offers critical knowledge the future translational applications.

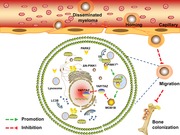

## 1 Introduction

Multiple myeloma (MM) is the second most common hematological malignancy in the United States accounting for an estimated 32 000 new diagnoses and 12 960 deaths in 2019 alone. MM remains an incurable disease and nearly all myeloma patients will eventually relapse from all conventional therapies, demonstrating the urgent need for a better understanding of the cellular and molecular mechanisms driving the pathogenesis and progression of this disease. Recently, reprogramming of energy metabolism has emerged as an additional hallmark of cancer.^1^ However, how energy metabolism has been reprogrammed and how alterations in metabolism contribute to myeloma pathogenesis and progression have thus far remained uncharacterized.

Mitochondria are the “powerhouse” of the cell and the center of energy metabolism. Studies over the past half‐century have implicated mitochondrial dysfunction in development of neurodegenerative diseases, diabetes, cellular aging, and cardiovascular diseases.^2^ Additionally, a growing body of evidence suggests that mitochondrial dysregulation is linked to cancer development and progression, and has a strong impact on the degree of cancer invasiveness and metastasis.^3^

Mitochondria form a comprehensive network and can rapidly adapt to meet the metabolic needs of cells by shifting the balance between fission and fusion, mitochondrial biogenesis, and mitophagy. This balance varies substantially among diverse eukaryotic lineages. In general, when there is an increased metabolic demand, mitochondria undergo biogenesis and fusion; on the other hand, a decrease in metabolic demand results in the removal of superfluous mitochondria via the process of fission and mitophagy.

Mitophagy, the process of mitochondrial autophagy, is induced by mitochondrial membrane depolarization or changes in mitochondrial DNA (mtDNA).^4^ Mitophagy protects against the release of proapoptotic proteins, generation of toxic reactive oxygen species (ROS), and futile hydrolysis of adenosine triphosphate (ATP) by aged, damaged, and depolarized mitochondria.[qv: 4c] The role of mitophagy in cancer pathogenesis is currently an area of active investigation, but the findings are varied. For instance, some studies have suggested that a decrease in mitophagy leads to an increase of free radical production and genetic instability,^5^ thus, favoring the development of cancer. Other studies have found that increased mitophagy protects cancer cells from apoptosis,^6^ thus promoting cancer cell survival and proliferation. The most likely explanation for these divergent findings is that mitophagy's role in cancer pathogenesis is cell‐type specific.[qv: 3b]

Various genes and molecules have been implicated in mitophagy. PTEN‐induced putative kinase 1 (PINK1)—a mitochondrial serine/threonine kinase—and Parkin/parkin RBR E3 ubiquitin protein ligase (PARK2)—an E3 ubiquitin ligase—act as master regulators of mitophagy.^7^ PINK1–PARK2‐dependent mitophagy is the most well characterized mitophagic pathway. During mitophagy, PINK1 is stabilized on the outer mitochondrial membrane, facilitating PARK2 recruitment.^8^ PARK2 then ubiquitinates and promotes degradation of several outer mitochondrial membrane proteins, leading to mitochondrial aggregation, clustering, and phagophore nucleation. Phagophores are targeted to the mitochondria via specific receptors such as LC3B and the sequestered mitochondria are then degraded by fusion to lysosomes.

In the current study, we determined the role of PINK1–PARK2‐dependent mitophagy in myeloma cell spreading and progression.

## 2 Results

### 2.1 PINK1‐Dependent Mitophagy Is Suppressed in Multiple Myeloma Cells and Correlates with Clinical Outcomes in Patients with Multiple Myeloma

**Figure 1:**
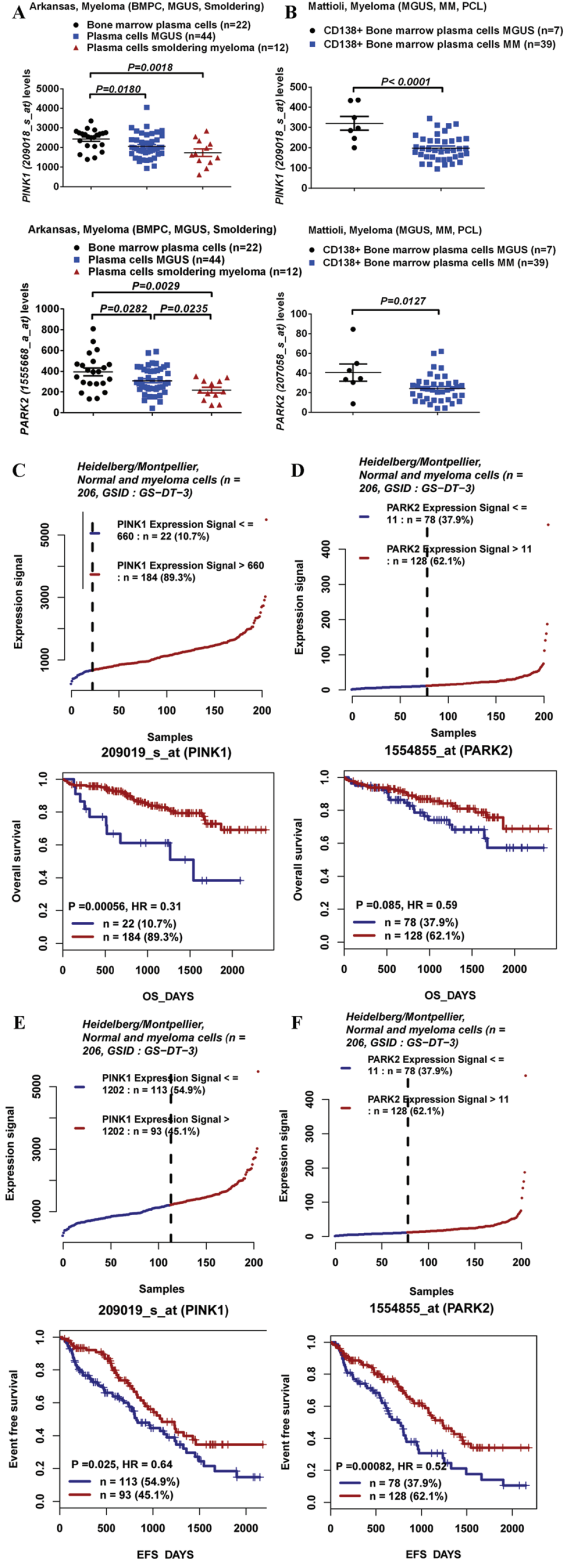
Level of PINK1‐dependent mitophagy markers (i.e., *PINK1* and *PARK2* expression) and correlation of PINK1‐dependent mitophagy with clinical outcomes in patients with MM. A,B) *PINK1* and *PARK2* expression levels in MGUS plasma cells and myeloma cells in the A) Arkansas and B) Mattiloli datasets. Expression levels were presented as scatter plot and were compared using an unpaired Student's *t*‐test. C,D) Kaplan–Meyer analysis of overall survival in the Heidelberg/Montpellier dataset basing on the expression of *PINK1* and *PARK2* in CD138^+^ cells of myeloma patients. Survival analysis was performed using a log‐rank test. High and low *PINK1* expression (ID: 209019_s_at) was defined using a cut‐off of 660. High and low *PARK2* expression (ID: 1554855_at) was defined using a cut‐off of 11. E,F) Kaplan–Meyer analysis of event‐free survival in the Heidelberg/Montpellier dataset basing on the expression of *PINK1* and *PARK2* in CD138^+^ cells of myeloma patients. Survival analysis was performed using a log‐rank test. High and low *PINK1* expression (ID: 209019_s_at) was defined using a cut‐off of 1202. High and low *PARK2* expression (ID: 1554855_at) was defined using a cut‐off of 11.

We first investigated the levels of the PINK1‐dependent mitophagy markers, i.e., *PINK1* and *PARK2* expression and the correlation between their expression levels and clinical outcomes in MM patients. Using three publicly available datasets, we analyzed the mRNA level of *PINK1* and *PARK2* in bone marrow CD138^+^ cells of patients with monoclonal gammopathy of undetermined significance (MGUS) or MM. We found that the levels of PINK1 and PARK2 were consistently reduced in patients with MM compared to patients with MGUS in datasets analyzed (**Figure** **1**A,B; Figure S1A,B, Supporting Information). We then analyzed the correlation between level of PINK1‐dependent mitophagy and overall survival in myeloma patients. We interrogated microarray data from four large publicly available datasets (GSID: GD‐DT‐3, Heidelberg/Montpellier dataset;^9^ GSID: GS‐DT‐14, Arkansas dataset;^10^ GSID: GS‐DT‐52, Mulligan dataset;^11^ GSID: GS‐DT‐59, Fonseca dataset^12^) representing a total of 1028 patients. As shown in Figure 1C–F using one of these datasets (GSID: GD‐DT‐3), we found that a reduced level of PINK1‐dependent mitophagy (represented by lower levels of *PINK1* and *PARK2* expression) correlated with worse overall and event free survival in patients with MM. Similar correlations were observed in the Arkansas dataset (Figure S1C,D, Supporting Information). These data indicate an important role of PINK1‐dependent mitophagy in MM pathogenesis.

### 2.2 PINK1‐Dependent Mitophagy Regulates Myeloma Cells' Transwell Migration In Vitro

We next set out to determine how PINK1‐dependent mitophagy contributes to myeloma pathogenesis. To this end, we used three different approaches to induce PINK1‐dependent mitophagy (treatment with carbonylcyanide‐m‐chlorophenylhydrazone (CCCP), salinomycin, or overexpression of PINK1), and then examined the effects of increased mitophagy on myeloma cell proliferation, survival, and migration in vitro.

First, we treated seven myeloma cell lines (MM.1S, MM.1R, OPM1, RPMI8226, RPMI8226/DOX, NCIH929, and U266) with CCCP which disrupts mitochondrial membrane potential and is commonly used to selectively induce mitophagy.[qv: 4b,13] As expected, CCCP induced mitophagy as demonstrated by increased mitochondria membrane depolarization (green JC‐1 aggregates measured by MitoProbe, right lower quadrant in Figure S2A in the Supporting Information), increased mRNA and protein expression of *PINK1* and *PARK2* (Figure S2B,C, Supporting Information), and the conversion of LC3B‐I to LC3B‐II (Figure S2C, Supporting Information). Next, we treated with salinomycin, an antibacterial and coccidiostat agent, which is a known inducer of mitophagy.^14^ Compared to CCCP, salinomycin causes more gentle disruption of mitochondrial functions (Figure S2A, Supporting Information), but did result in increased mRNA and protein expression of *PINK1* and *PARK2* (Figure S2B,C, Supporting Information) and the conversion of LC3B‐I to LC3B‐II (Figure S2C, Supporting Information). Furthermore, CCCP or salinomycin treatment inhibited the oxygen consumption rate (OCR) and extracellular acidification rate (ECAR), and significantly reduced ATP production and spare respiratory capacity (Figure S2D, Supporting Information), consistent with the induction of mitophagy,

To further confirm the induction of mitophagy by CCCP or salinomycin treatment, we transduced MM cells with MitoTrack vector (red color) and LC3B‐EGFP vector (green color) to observe the fusion of mitochondria with lysosomes (yellow color on the merged image) using confocal microscopy. As shown in Figure S2E in the Supporting Information, both CCCP and salinomycin induced mitophagy. The induction of mitophagy was further confirmed by visualization of the engulfment/fusion of mitochondria with lysosomes by transmission electron microscope (TEM) (Figure S2F, Supporting Information).

**Figure 2:**
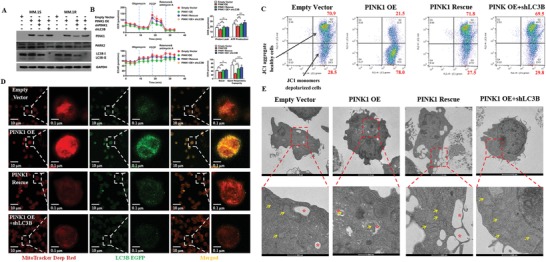
Overexpression of PINK1 induces mitophagy. A) Western blot analysis of PINK1, PARK2, and LC3B protein levels in MM cells under different gene transductions. MM cells were transduced with empty control vector, PINK1 overexpressing vector (PINK1 OE), PINK1 overexpressing vector followed by transduction with PINK1 specific shRNA (PINK1 rescue), or PINK1 overexpressing vector followed by transduction with LC3B specific shRNA (PINK1 OE + shLC3B). B) Mitochondrial respiration. OCR and ECAR were measured using seahorse XF. *X*‐axis represented total running time, while *Y*‐axis represented OCR or ECAR (for a total of 25 000 MM.1S myeloma cells). Proton leak, ATP production and spare respiratory capacity were calculated. Data represented mean ± SEM, *n* = 4–6. *p < 0.05, **p < 0.01, and ***p < 0.001. C) PINK1 overexpression induces mitochondrial membrane depolarization. Mitochondrial membrane depolarization was measured by JC1 MitoProbe. MM cells were transduced with empty control vector, PINK1 OE vector, PINK1 rescue, or PINK1 OE + shLC3B. One of the three representative experiments was shown. Upper right quadrant represented cells with normal mitochondrial potential and right lower quadrant represented cells having depolarized mitochondria. D) Confocal microscopy of the fusion of mitochondria and lysosome. After transduction with various vectors mentioned above, the cells were then transduced with MitoTracker (deep red color) and LC3B‐eGFP (green color) constructs. The fusion of mitochondria and lysosomes revealed as yellow color on the merged images. E) TEM imaging of the fusion of mitochondria and lysosome. Mitophagy (i.e., the fusion of mitochondria, represented as red asterisks, with lysosome, represented by yellow arrows) was detected by TEM in the cells described above.

Finally, we induced genetic overexpression of PINK1 in MM cell lines which has previously been shown to induce mitophagy in cancer cell lines.^15^ Seven MM cell lines were transduced with a lentiviral vector expressing the PINK1 gene or an empty control lentiviral vector. To further confirm the effect of PINK1 on mitophagy, the PINK1 overexpressing MM cells were transduced with PINK1‐ or LC3B‐specific shRNAs (**Figure** **2**A). PINK1 overexpression impaired mitochondrial respiration either under basal condition or after FCCP treatment as demonstrated by the decreased ATP production and the reduced spare respiratory capacity (Figure 2B), consistent with reduced mitochondrial mass. Furthermore, PINK1 overexpression induced mitochondrial membrane depolarization as measured by JC‐1 MitoProbe (Figure 2C). PINK1‐specific shRNA knockdown or LC3B‐specific shRNA knockdown restored mitochondrial respiration/mass, and abrogated the mitochondrial membrane depolarization induced by PINK1 overexpression (Figure 2B,C). Confocal microscopy (Figure 2D) and TEM (Figure 2E) further validated the induction of mitophagy by PINK1 overexpression and its reversal with PINK1‐ or LC3B‐specific shRNA knockdown. These data demonstrate the induction of mitophagy by PINK1 gene overexpression in myeloma cells.

**Figure 3:**
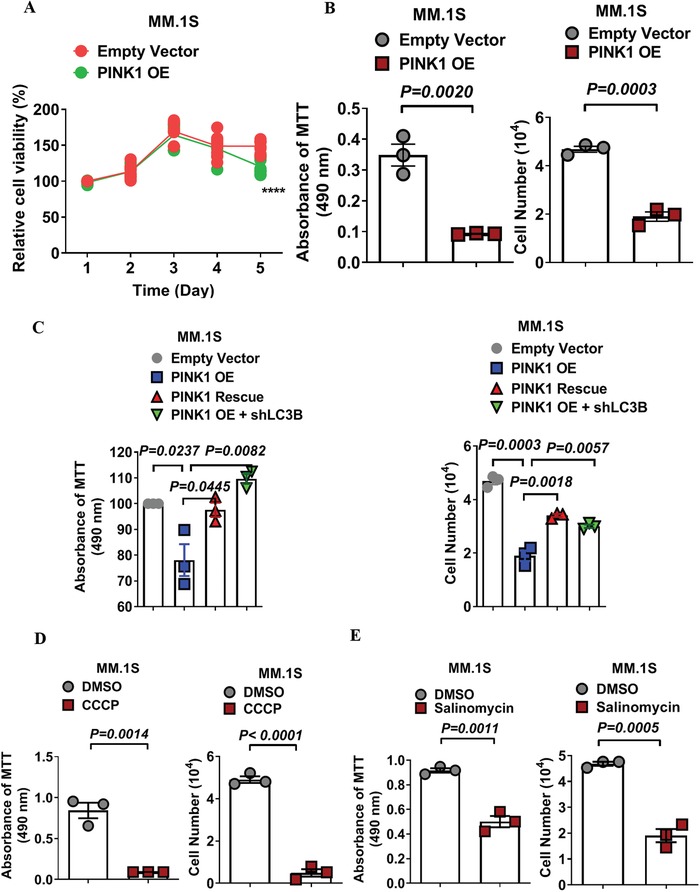
PINK1‐dependent mitophagy inhibits myeloma cells' transwell migration in vitro. A) The effect of PINK1 overexpression on myeloma cell proliferation and cell transwell migration. MM cells were transduced with control vector (EV) and PINK1 OE vector. Cell proliferation was determinate by MTT assay. Data represented mean ± SEM, *n* = 6. B) The effect of PINK1 overexpression on myeloma cell migration. MM cells were transduced with empty control vector or PINK1 OE, and cell transwell migration assay was performed. *Y*‐axis represented migrated cells in the lower chamber by MTT absorbance (left panel) or by cell counts (right panel). C) PINK1‐dependent mitophagy inhibits myeloma cells' transwell migration. MM.1S cells were transduced with empty vector, PINK1 OE, PINK1 rescue, or PINK1 OE + shLC3B. Cell migration was measured by transwell assay. *Y*‐axis represented migrated cells in the lower chamber by MTT absorbance (left panel) or by cell counts (right panel). Data represented mean ± SEM, *n* = 4–6. D) CCCP treatment reduces transwell migration of myeloma cells. MM.1S cells were treated with DMSO or CCCP (5 × 10^−6^ m) for 48 h and transwell cell migration was measured by MTT absorbance (left panel) or by cell counts (right panel). E) Salinomycin treatment inhibits cell transwell migration of myeloma cells. MM.1S cells were treated with DMSO or salinomycin (2.5 × 10^−6^ m) for 48 h. Cell migration was measured as described in CCCP. Data represented mean ± SD, *n* = 3.

We then examined the downstream effects of these three different mitophagy induction treatments. Overexpression of PINK1 did not affect myeloma cell viability or proliferation except in one MM cell line (MM.1S) where a modest inhibition of cell proliferation was noted in PINK1 OE at day 5 of culture (**Figure** **3**A; Figure S3A, Supporting Information). Overexpression of PARK2 also did not generally affect the viability or proliferation of MM cells (Figure S3B, Supporting Information). Furthermore, we found that PINK1 overexpression had minimal effects on myeloma cell cycling (Figure S3C, Supporting Information) or on the induction of apoptosis (Figure S3D, Supporting Information). Interestingly though, when we measured myeloma cell transwell migration at day 4 after gene transduction when myeloma cell viability was not affected, we found that overexpression of PINK1 significantly inhibited the transwell migration of all MM cell lines as measured by MTT absorbance or by cell counts (Figure 3B).

Compared to cell proliferation and apoptosis, the effect of PINK1 overexpression on myeloma cell migration was more profound, suggesting that PINK1‐dependent mitophagy predominantly affects myeloma cell migration. To confirm that this migration inhibition was indeed mediated by increased mitophagy, we knocked down LC3B using LC3B‐specific shRNA which restored the myeloma cell's transwell migration (Figure 3C; Figure S4A,B, green dots, Supporting Information). PINK1 knockdown by shRNA also restored the transwell migration of myeloma cells (Figure 3C; Figure S4A,B, red dots, Supporting Information).

Similar to PINK1 overexpression, treatment with CCCP or salinomycin significantly reduced myeloma cell migration in vitro (Figure 3D,E; Figure S5A–F, Supporting Information). Unlike PINK1 genetic overexpression, treatment with CCCP or salinomycin significantly inhibited myeloma cell survival and proliferation (Figure S5G,H, Supporting Information). This inhibitory effect on myeloma cell proliferation seen with CCCP and salinomycin but not with PINK1 gene overexpression is likely related to the dramatic and rapid burst of mitophagy induction with the pharmacological approach.

### 2.3 PINK1‐Mediated Mitophagy Regulates Myeloma Cell Early Homing and Tumorigenesis In Vivo

**Figure 4:**
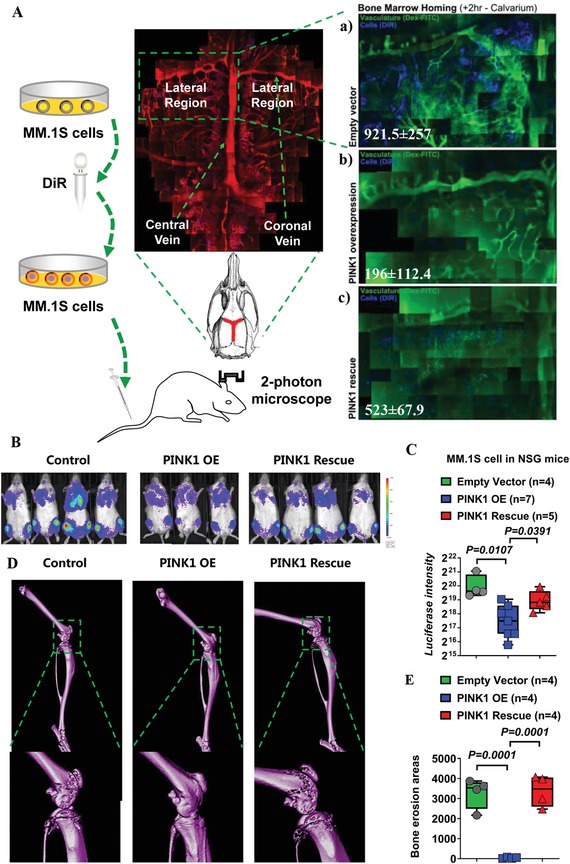
PINK1‐dependent mitophagy inhibits myeloma cell homing and tumor development in vivo. A) Intravital imaging for the measurement of myeloma cell homing and migration. MM.1S cells were transduced by empty vector, PINK1 overexpressing vector (OE), or PINK1 overexpressing vector followed by transduction with PINK1 specific shRNA. The cells were labeled with DiR fluorescent dye and injected via tail vein into NSG mice (0.5 × 10^6^ cells per mouse). A confocal microscopy was used to scan the mouse calvarium including the central sinus and the surrounding bone marrow cavities. High‐resolution images were obtained through the intact mouse skull and captured with Leica LAS‐AF software. DiR^+^ myeloma cells observed in the vasculature and in the bone marrow space were visualized at 2 h after cell injection. Green: Dex‐FITC vasculature. Blue: DiR^+^ myeloma cells. a) MM cells transduced with control vector. b) MM cells transduced with PINK1 OE vector. c) MM cells transduced with PINK1 OE vector followed by PINK1 shRNA (PINK1 rescue). One of the three representative experiments was shown. B–E) PINK1‐dependent mitophagy regulated myeloma cell in vivo tumorigenesis. MM cells were transduced with control vector, PINK1 OE vector, or PINK1 OE + PINK1 shRNA (PINK1 rescue), and injected i.v. into NSG mice. B) Forty‐five days later, tumor burden was measured by luciferin bioluminescence imaging. C) Statistical analysis of luciferin bioluminescence intensity in NSG mice xenografted with MM cells transduced with empty vector, PINK1 OE vector, or PINK1 rescue vector. Data represented mean ± SEM, *n* = 4–7. D) Bone structure was measured by micro‐CT. E) Statistical analysis of bone erosion in NSG mice xenografted with MM cells transduced with empty vector, PINK1 OE vector, or PINK1 rescue vector. Data represented mean ± SEM, *n* = 4–7.

Myeloma cells are constantly moving in and out of the bone marrow during myeloma development, making cell homing and migration critical for myeloma tumorigenesis and progression. We performed single‐cell resolution intravital imaging in real time to determine the effects of PINK1‐dependent mitophagy on cell migration and homing in vivo. MM.1S cells were transduced with empty control lentiviral vector, PINK1‐overexpressing lentiviral vector (PINK1 OE), or PINK1 overexpression followed by PINK1 shRNA knockdown (PINK1 rescue). These cells were then labeled with DiR dye and injected intravenously into NOD/SCID IL‐2gamma^null^ (NSG) mice. DiR^+^ MM cells in the calvarium vasculature (the central vessel, parasagittal sinusoids, and the lateral region) and the bone marrow space were measured at 2 h after cell injection using confocal microscopy as previously described (**Figure** **4**A).^16^ Overexpression of PINK1 demonstrated a trend to suppress myeloma cell homing/migration to the calvarium at 2 h after cell injection (Figure 4A‐a,b), which was attenuated by a PINK1‐specific shRNA (Figure 4A‐c) (the full images are included in Figures S8–S10 and the video recordings are provided in Videos S11–S13 in the Supporting Information).

To further determine the role of mitophagy in myeloma development, MM cells transduced with control vector, PINK1 OE vector or PINK1 rescue were injected intravenously into NSG mice. Forty‐five days later, tumor development was measured by luciferin bioluminescence imaging (Figure 4B,C), and microcomputed tomography (micro‐CT) imaging (Figure 4D,E). As shown in Figure 4B–E, mice injected with PINK1 overexpressing MM cells had significantly less tumor burden and bone destruction, compared to mice injected with MM cells transduced with control vector. Furthermore, PINK1‐specific shRNA knockdown abrogated the beneficial effects of PINK1 overexpression. These data demonstrate the important role of PINK1‐dependent mitophagy in myeloma cell migration and the pathogenesis of myeloma in vivo.

### 2.4 Treatment with Salinomycin Demonstrates Effective In Vivo Antimyeloma Activity in a Murine Myeloma Xenograft Model

**Figure 5:**
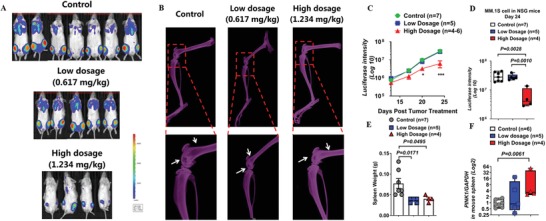
Salinomycin inhibited myeloma development and attenuated bone erosion in vivo. A) Salinomycin treatment inhibited myeloma tumor growth. MM cells stably expressing luciferase reporter were injected i.v. into sublethally (1.5 Gy) irradiated NSG mice. When tumors were established, the mice were treated with PBS control buffer or salinomycin (0.617 or 1.234 mg kg^−1^) by i.p. injection twice a week for 2 weeks followed by daily for additional 10 days. Bioluminescent imaging of the individual mice at the end of the experiment. B) Representative X‐ray images of bone lesions. White arrows represent bone lytic lesions. C) Tumor growth was monitored by bioluminescence imaging. D) Statistical analysis of tumor burden by bioluminescence imaging at the end of the experiment (*p < 0.05; ***p < 0.001). E) Spleen weight of NSG mice treated with control PBS buffer or salinomycin (0.617 or 1.234 mg kg^−1^) at the end of the experiment. F) Spleen *PINK1* mRNA expression level in mice treated with control PBS buffer or salinomycin (0.617 or 1.234 mg kg^−1^). Data represented mean ± SEM, *n* = 4–7.

We next tested if mitophagy can be targeted for the treatment of MM. We used salinomycin, a pharmacological agent known to selectively remove damaged mitochondrion without causing immediate, total mitochondria loss. Salinomycin was recently demonstrated to possess anticancer and anticancer stem cell effects in both preclinical models and in isolated case reports of advanced cancer patients.^17^ To assess salinomycin's efficacy in vivo, we injected MM.1S cells stably expressing luciferase reporter intravenously into 1.5 Gy total body‐irradiated NSG mice. When tumors were established, the mice were treated with vehicle control or with salinomycin (0.617 or 1.234 mg kg^−1^) i.p. twice weekly for 2 weeks then daily for an additional 10 days. The dose was calculated according to the dosage used in the clinical study (120 µg kg^−1^ in humans). Salinomycin treatment at 1.234 mg kg^−1^ resulted in significant decrease in tumor burden as demonstrated by bioluminescent imaging of luciferase expressing MM cells (**Figure** **5**A–D) and significant decrease in bone destruction as measured by X‐ray (Figure 5B). Furthermore, splenic involvement of myeloma cells was also reduced with salinomycin treatment (Figure 5E). To determine if salinomycin induced mitophagy in our in vivo myeloma xenograft model, we measured PINK1 expression in splenocytes. As shown in Figure 5F, salinomycin induced upregulation of PINK1, and the degree of PINK1 upregulation correlated with its in vivo antimyeloma activity.

### 2.5 Mitophagy Induction Is Associated with Increased Expression of Mps One Binder Kinase Activator (MOB1B) and Downregulation of Yes‐Associated Protein (YAP)/Transcriptional Co‐Activator with PDZ‐Binding Motif (TAZ) in Myeloma Cells

**Figure 6:**
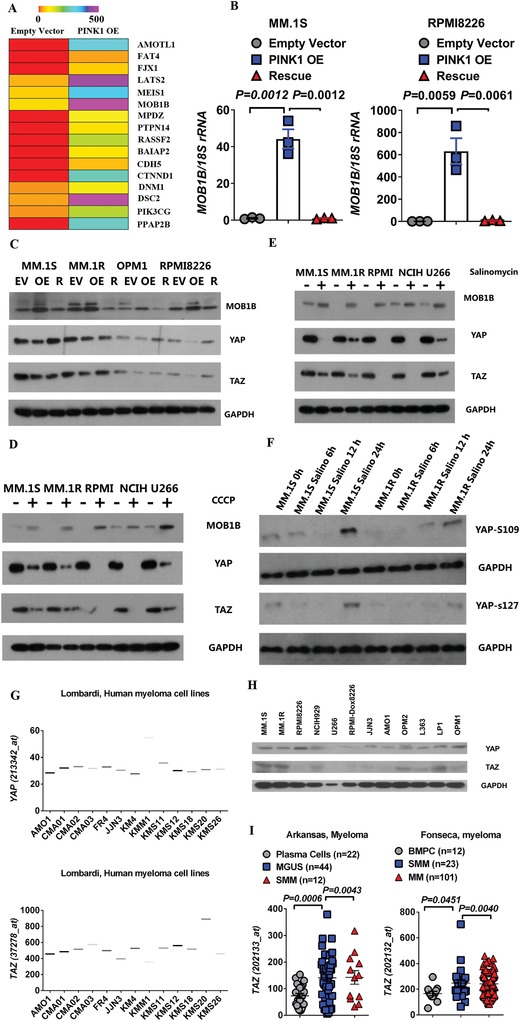
PINK1 overexpression upregulates MOB1B of Hippo pathway. A) qPCR array. MM.1S cells transduced with control vector or PINK1 OE vector were subject to PCR array analysis. Changes in gene expression were shown in the heatmap. B) PINK1 upregulates MOB1B mRNA expression. MOB1B mRNA expression in MM cells transduced with control vector (EV), PINK1 OE, or PINK1 OE + shPINK1 (rescue or R). C) MOB1B, YAP, and TAZ expression in MM cells transduced with control vector (EV), PINK1 OE, or PINK1 OE + shPINK1 (R) as measured by western blot analysis. D) MOB1B, YAP, and TAZ expression in MM cells treated with DMSO control (−) or 5 × 10^−6^ m CCCP (+) for 48 h. E) MOB1B, YAP, and TAZ expression in MM cells treated with DMSO control (−) or 2.5 × 10^−6^ m salinomycin for 48 h (+). F) Salinomycin treatment led to phosphorylation of YAP. MM.1S and MM.1R cell lines were treated with 2.5 × 10^−6^ m salinomycin for 0, 6, 12, and 24 h and p‐YAP‐S109 and p‐YAP‐S127 were measured by western blot. G) YAP and TAZ mRNA expression in 13 myeloma cell lines in the Lombardi microarray dataset. H) Level of YAP and TAZ protein expression in 12 MM cell lines measured by Western blot analysis. I) Upregulation of TAZ expression in myeloma patients. TAZ expression in myeloma CD138^+^ cells in two publicly available microarray datasets as compared to normal plasma cells, MGUS CD138^+^ cells, and smoldering myeloma CD138^+^ cells.

To dissect the molecular pathways underlying mitophagy‐induced inhibition of myeloma cell migration and homing, we performed an adhesion and metastasis‐specific qPCR array in MM.1S cells transduced with empty vector (EV) or PINK1 OE vector (**Figure** **6**A). We found that both large tumor suppressor homologue (LATS) mRNA and the tumor suppressor MOB1B mRNA were highly upregulated in PINK1 overexpressing MM cells (Figure 6A). LATS and MOB1B are the core components of the mammalian Hippo pathway.^18^ Upon Hippo pathway activation, the paralogous transcriptional co‐activators YAP and TAZ become phosphorylated and undergo degradation. YAP and TAZ play an important role in apoptosis, cell migration, and stem cell maintenance.^18^, ^19^

We performed qPCR and western blot analyses to validate our findings in MM.1S and other MM cell lines. PINK1 overexpression upregulated the expression of MOB1B at both mRNA and protein levels; and PINK1‐specific shRNA attenuated the MOB1B expression to control level in all MM cell lines tested (Figure 6B,C; Figure S6, Supporting Information). Furthermore, as shown in Figure 6C, PINK1 overexpression significantly downregulated YAP and TAZ expression, and specific PINK1 knockdown abolished these effects. Similarly, treatment with CCCP or salinomycin led to the upregulation of MOB1B and the downregulation of YAP and TAZ in all MM cell lines tested (Figure 6D,E). Additionally, treatment with salinomycin for 24 h led to enhanced phosphorylation of YAP at pYAP‐S109 and pYAP‐S127 (Figure 6F).

**Figure 7:**
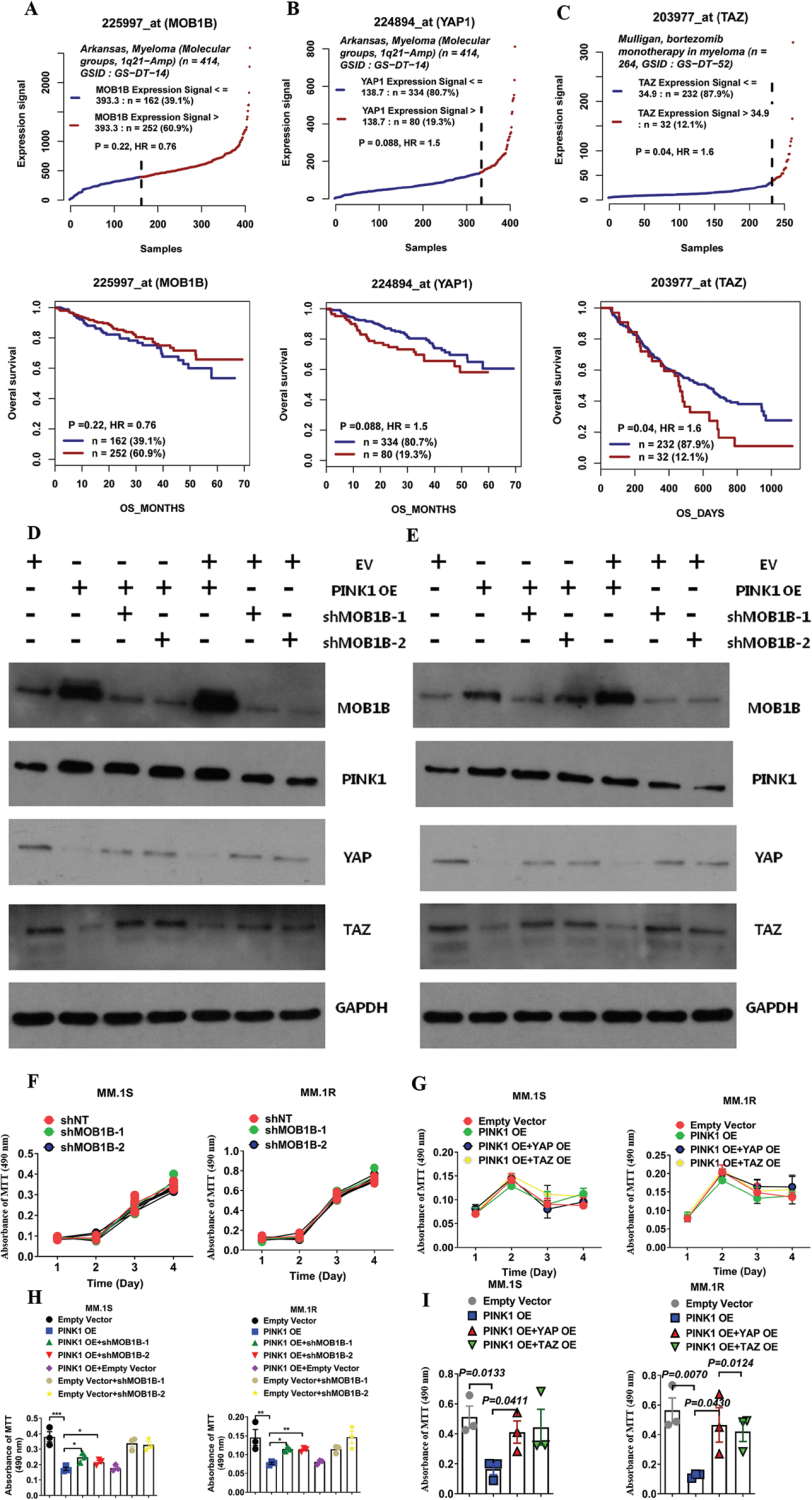
MOB1B and YAP/TAZ pathway plays an important role in PINK1‐dependent mitophagy‐mediated inhibition of myeloma cell migration. A) Higher expression of MOB1B demonstrated a trend for better overall survival in Arkansas myeloma microarray dataset. B,C) Kaplan–Meyer analysis of overall survival in the Arkansas and Mulligan datasets basing on the expression of B) YAP or C) TAZ in CD138^+^ cells of myeloma patients. Survival analysis was performed using a log‐rank test. D,E) MOB1B specific shRNA knockdown restored YAP/TAZ expression. D) PINK1 overexpressing MM.1S cells and E) PINK1 overexpressing MM.1R cells were transduced with two different MOB1B shRNA. MOB1B, YAP, and TAZ expression was measured by Western blot analysis. F) MOB1B specific shRNA knockdown did not affect myeloma cell proliferation. MM.1S cells (left panel) and MM.1R cells (right panel) were transduced with two different MOB1B shRNA and cell proliferation was measured by MTT assay. G) Overexpression of YAP or TAZ did not affect myeloma cell proliferation. PINK1 overexpressing MM.1S cells (left panel) and PINK1 overexpressing MM.1R cells (right panel) were transduced with YAP overexpressing vector or TAZ overexpressing vector. Cell proliferation was measured by MTT assay. H) MOB1B specific shRNA knockdown restored myeloma cell transwell migration that was inhibited by PINK1 overexpression. PINK1 overexpressing MM.1S cells (left panel) and PINK1 overexpressing MM.1R cells (right panel) were transduced with two different MOB1B shRNA. Myeloma cell migration was measured by transwell assay. I) Overexpression of YAP or TAZ restored myeloma cell transwell migration that was inhibited by PINK1 overexpression. PINK1 overexpressing MM.1S cells (left panel) and PINK1 overexpressing MM.1R cells (right panel) were transduced with YAP overexpressing vector or TAZ overexpressing vector. Myeloma cell migration was measured by transwell assay.

We examined the expression level of YAP/TAZ in myeloma cells. To this end, we first downloaded the gene expression data of YAP/TAZ on over 13 myeloma cell lines from the publicly available Lombardi microarray dataset and found that both YAP and TAZ genes were detectable (Figure 6G). Western blot analyses of 12 different myeloma cell lines demonstrated the expression of YAP and TAZ protein in the majority of myeloma cell lines tested (Figure 6H). Additionally, TAZ mRNA was significantly upregulated in the bone marrow CD138^+^ cells of patients with MM compared to that in normal plasma cells or in patients with MGUS (Figure 6I). We further analyzed the correlation between the expression of MOB1B, YAP and TAZ and clinical outcomes of patients with MM. We found that increased level of MOB1B demonstrated a trend for better overall survival in patients with myeloma (**Figure** **7**A). In contrast, the higher expression of YAP or TAZ correlated with decreased overall survival in patients with MM (Figure 7B,C).

To further confirm whether MOB1B has a direct role in PINK1‐mediated myeloma cell migration, we knocked down MOB1B using MOB1B‐specific shRNA in the PINK1 overexpression MM cells and examined its effects on the expression of YAP and TAZ and myeloma cell transwell migration. MOB1B‐specific knockdown upregulated YAP and TAZ expression (Figure 7D,E). MOB1B shRNA did not affect MM cell proliferation (Figure 7F). However, MOB1B‐specific shRNA knockdown restored at least in part myeloma cell transwell migration that was attenuated by PINK1 overexpression (Figure 7H). Similarly, we transduced PINK1 overexpressing MM cells with YAP‐ or TAZ‐expressing lentiviral vector. YAP or TAZ overexpression did not affect myeloma cell proliferation (Figure 7G). Overexpression of YAP or TAZ abrogated the effects of PINK1 on myeloma cell migration (Figure 7I). These data demonstrate a critical role of MOB1B‐YAP/TAZ pathway in PINK1‐mediated myeloma cell migration.

### 2.6 *Pink1* Deletion in X‐Box Binding Protein‐1s (*XBP‐1s*) Transgenic Mice Demonstrates Faster Myeloma Development and Progression

**Figure 8:**
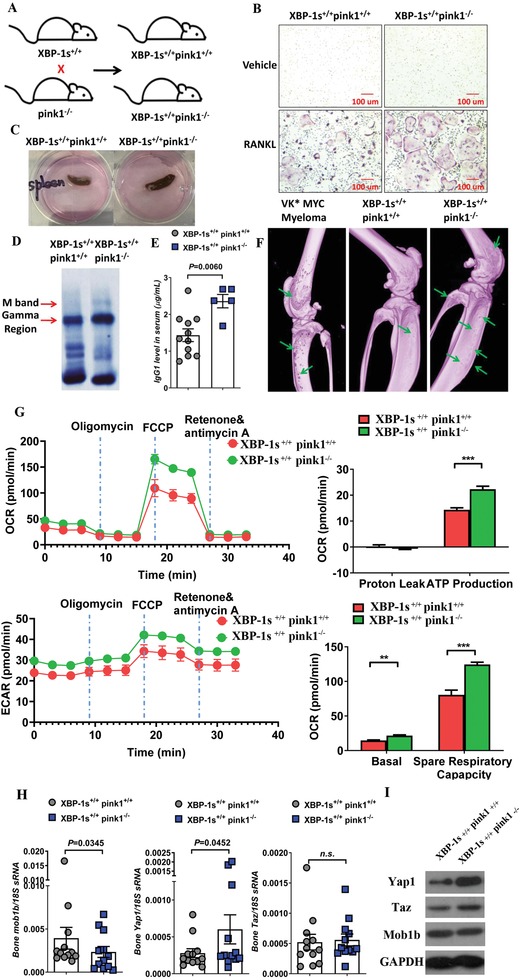
Pink1 deletion accelerates myeloma development and progression in XBP‐1s transgenic mice. A) Schematic diagram of crossbreeding *XBP‐1s^+/+^* mice with *pink1^−/−^* mice to generate the *XBP‐1s^+/+^ pink1^−/^* ^−^ mice. B) Bone marrow osteoclast differentiation from *XBP‐1s^+/+^ pink1^−/−^* and *XBP‐1s^+/+^ pink1^+/+^* mice. Representative TRAP staining after 6 day of differentiation. C) Representative image of mouse spleen from *XBP‐1s^+/+^ pink1^+/+^* (left) and *XBP‐1s^+/+^ pink1^−/−^* (right) mice. D,E) Serum protein electrophoresis with D) the gamma protein and M spike indicated and E) the serum IgG1 level from *XBP‐1s^+/+^ pink1^+/+^* and *XBP‐1s^+/+^ pink1^−/−^* mice. Data represent mean ± SEM. *p < 0.05. F) Micro‐CT imaging of the tibiae and femur from VK*MYC myeloma (left), *XBP‐1s^+/+^ pink1^+/+^* (middle), and *XBP‐1s^+/+^ pink1^−/−^* (right) mice. Green arrows indicated bone punch lytic lesions. G) Mitochondrial respiration by seahorse assay using bone marrow cells harvested from *XBP‐1s^+/+^ pink1^+/+^* and *XBP‐1s^+/+^ pink1^−/−^* mice. H) Bone marrow Mob1b, Yap1, and Taz mRNA expression harvested from *XBP‐1s^+/+^ pink1^+/+^* and *XBP‐1s^+/+^ pink1^−/−^* mice measured by RT‐PCR. I) Protein expression of Mob1b, Yap1, and Taz in *XBP‐1s^+/+^ pink1^+/+^* and *XBP‐1s^+/+^ pink1^−/−^* mice was measured by Western blot.

To further determine the role of PINK1‐dependent mitophagy in myeloma pathogenesis in vivo, we crossbred *pink1* ^−/−^ mice with Eµ‐XBP‐1s transgenic mice (*XBP‐1s* ^+/+^) and generated the *XBP‐1s* ^+/+^/*pink1* ^−/−^ mice (**Figure** **8**A). XBP‐1 spliced isoform (XBP‐1s) is a transcription factor governing unfolded protein/endoplasmic reticulum (ER) stress response and plasma‐cell development.^20^ Eµ‐XBP‐1s transgenic mice exhibit a phenotype similar to human MGUS/MM disease:^21^ specifically, the mice develop the MGUS phenotype which spontaneously transforms to MM around age 10–14 months.^22^ We found that *XBP‐1s^+/+^/pink1^−/−^* mice had accelerated levels of osteoclast differentiation even at 8–10 weeks old: the size of osteoclasts was over threefold bigger in *XBP‐1s^+/+^/pink1^−/−^* mice compared to that in *XBP‐1s^+/+^/pink1^+/+^* littermate controls (Figure 8B). When we sacrificed the mice at 10 months old, compared to the *XBP‐1s^+/+^/pink1^+/+^* littermates *XBP‐1s^+/+^/pink1^−/−^* mice exhibited enlarged spleen (Figure 8C, ≈160 mg in *XBP‐1s^+/+^/pink1^−/−^* mice vs ≈90 mg in *XBP‐1s^+/+^/pink1^+/+^* mice) and doubling of the number of bone marrow CD38^+^ plasma cells (0.08% in *XBP‐1s^+/+^/pink1^−/−^* mice vs 0.04% in *XBP‐1s^+/+^/pink1^+/+^*mice) and the spleen CD38^+^ plasma cells (0.23% in *XBP‐1s^+/+^/pink1^−/−^* mice vs 0.14% in *XBP‐1s^+/+^/pink1^+/+^* mice). *XBP‐1s^+/+^/pink1^−/−^* mice showed the development of M protein and higher levels of gamma protein (Figure 8D) and IgG (Figure 8E) and the presence of bone lytic lesions (Figure 8F). Furthermore, bone marrow cells from *XBP‐1s^+/+^/pink1^−/−^* mice showed increased mitochondrial respiration/mass (Figure 8G), downregulated Mob1b mRNA expression, and slightly increased Yap gene expression (Figure 8H). Western blot analysis revealed slight decrease of Mob1b and significant upregulation of Yap and Taz protein in *pink1* knockout mice (Figure 8I). These findings mirror what we observed with PINK1 overexpression in MM cells. These data demonstrate that *pink1* deletion in vivo suppresses mitophagy, inhibits Mob1b, and facilitates myeloma development.

### 2.7 Microenvironmental Deletion of *pink1* in C57BL/6 Mice Accelerates VK*MYC Myeloma Development

**Figure 9:**
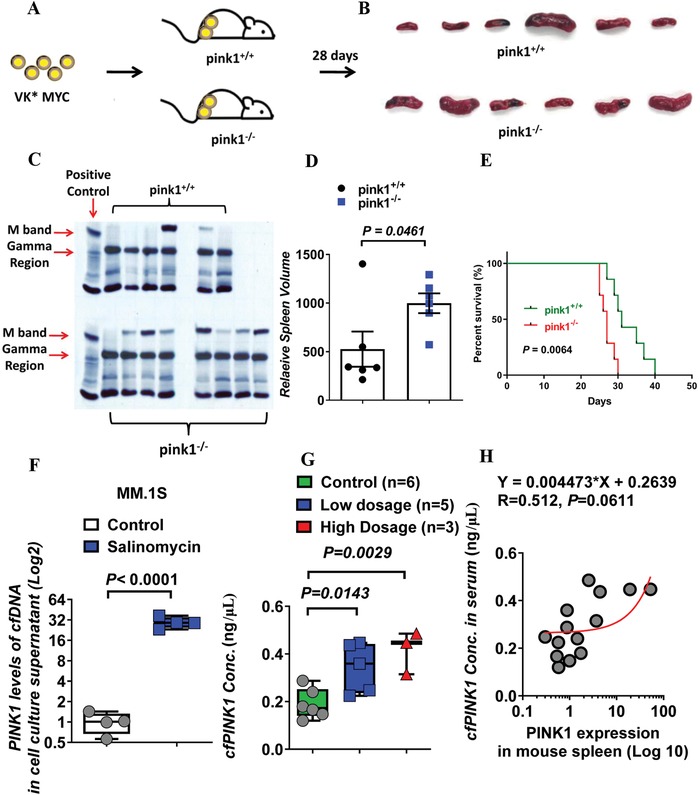
Pink1 deletion accelerates myeloma development and progression in syngeneic transplanted VK*MYC myeloma mice. A) Schematic diagram of injecting VK*MYC myeloma splenocytes into *pink1^−/−^* mice and *pink1^+/+^* littermate controls. B) Representative image of mouse spleen from *pink1^+/+^* (upper panel) and *pink1^−/−^* (lower panel) mice. C) Serum protein electrophoresis of sera from *pink1^+/+^* (upper panel) and *pink1^−/−^* (lower panel) mice. Serum from patients with myeloma was used as the positive control in the first lane. D) Relative spleen volume in mice with or without *pink1* deletion. E) Survival of *pink1^+/+^* and *pink1^−/−^* mice injected with VK*MYC myeloma cells. F–H) cfPINK1 DNA as biomarker for salinomycin treatment. F) cfPINK1 DNA was measured in cell culture medium treated with salinomycin or in plasmas of mice treated with DMSO, low dose (0.617 mg kg^−1^) salinomycin, or high dose (1.234 mg kg^−1^) salinomycin. H) Correlative analysis of spleen *PINK1* (log 10 transformation of relative ^△△^ct value, *X*‐axis) and cfPINK in mouse plasma (*Y*‐axis) after salinomycin treatment.

To determine whether PINK1‐dependent mitophagy in the microenvironment would affect myeloma development, the syngeneic transplantable VK*MYC myeloma mouse model was used.^23^ VK*MYC myeloma splenocytes were injected via tail vein into 10–12 week old, nonirradiated syngeneic *pink1* ^−/−^ knockout mice (on C57Bl/6 background) or *pink1* ^+/+^ C57Bl/6 littermates (**Figure** **9**A). Myeloma development was monitored by measurement of the M spike on serum protein electrophoresis. When the recipient mice were sacrificed at day 28, only 2 out of 6 (33.3%) *pink1* ^+/+^ C57Bl/6 recipients developed myeloma (Figure 9B,C). In contrast, 7 out of 8 (87.5%) *pink1* ^−/−^ recipient mice developed myeloma (Figure 9B,C). Additionally, *pink1* ^−/−^ recipient mice had higher spleen involvement (Figure 9D) and died earlier (Figure 9E). These data suggest that reduced levels of systemic PINK1‐dependent mitophagy facilitates myeloma development in vivo.

### 2.8 Cell‐Free PINK1 DNA Correlates with the Induction of Mitophagy and the In Vivo Antimyeloma Activity of Salinomycin

Because deletion of pink1 in myeloma cells or in the microenvironment promotes myeloma development (Figures 8 and 9), we wanted to test the utility of plasma cell‐free PINK1 DNA as a potential biomarker for mitophagy and for treatment response. Cell‐free DNAs (cfDNAs) are DNA fragments that are released from tumor cells or systemic cells and have been used as a less‐invasive biomarker in several cancers.^24^ Studies have suggested that genetic mutations and copy number changes in cfDNAs could be used to monitor treatment responses or evolution of tumors.^25^ To determine whether PINK1 can be released and detected as cfDNA, we measured cfPINK1 DNA in the culture medium of MM cells treated with CCCP or salinomycin. Treatment with CCCP or salinomycin caused an increased level of cfPINK1 DNA in the conditioned medium (Figure 9F; Figure S7A,B, Supporting Information). Additionally, we transduced MM cell lines with control vector, PINK1 OE vector, or PINK1 rescue. As shown in Figure S7C in the Supporting Information, cfPINK1 DNA expression level was increased in the conditioned media of PINK1 overexpressing cells and this level returned to baseline after treatment with PINK1 specific shRNA. We also measured plasma cfPINK1 DNA in mice treated with salinomycin as described in Figure 5, and found that cfPINK1 DNA levels was increased and correlated with salinomycin antimyeloma effectiveness in vivo (Figure 9G). Furthermore, mouse plasma cfPINK1 level correlated with the splenic PINK1 expression in the xenograft mice treated with salinomycin (Figure 9H). Additional studies are needed, but these results demonstrate the potential of using plasma cfPINK1 DNA as a biomarker for mitophagy and for salinomycin in vivo activity.

## 3 Discussion

This study uses genetic and pharmacologic approaches to provide direct evidence that PINK1‐dependent mitophagy plays a critical role in regulating myeloma migration, homing, and tumorigenesis. We have also shown that the level of PINK1‐dependent mitophagy correlates with myeloma patient's clinical outcomes and that noninvasive cfPINK1 level could be a useful marker for in vivo mitophagy measurement. Most importantly, this study demonstrates that mitophagy could potentially be targeted for the treatment of MM. Our findings shed new light on the molecular/metabolic mechanisms driving the pathogenesis and progression of MM, thus significantly advancing our understanding of MM.

Mitochondria are the essential site of aerobic energy production in eukaryotic cells. Maintaining a healthy population of mitochondria is essential to the proper function of cells. Autophagic delivery of mitochondria to lysosomes (mitophagy) is the major degradative pathway in mitochondrial turnover. Although long assumed to be a random process, accumulating lines of evidence have suggested that mitophagy is a selective process, which has been closely linked with nutrient deprivation,^26^ cell senescence,^27^ and aging.^28^ In normal physiology cells utilize mitophagy to get rid of damaged and dysfunctional mitochondria in an effort to maintain mitochondrial functional and genetic integrity, thus sustaining cellular homeostasis.

Several recent studies have demonstrated an important role of mitochondrial dysfunction in tumorigenesis and in tumor growth.^29^ The role of mitophagy in carcinogenesis is less characterized.^30^ A growing body of evidence suggests that mitophagy is pathogenic under some conditions.[qv: 5c] Excessive elimination of mitochondria by autolysosomes might be detrimental to cellular self‐renewal. However, some have argued that mitophagy was intimately involved in the survival and death of cells through the elimination of damaged and nonfunctional mitochondria, consequently preventing mitochondrial dysfunction, oxidative stress, and cell death. Our present study unequivocally demonstrates that reduced level of mitophagy contributes to MM development and pathogenesis in large part by enhancing the migration and spreading of myeloma cells.

The cellular and molecular events that suppress mitophagy in myeloma cells remain to be determined. Myeloma cells and plasma cells are unique in their ability to secrete large amounts of monoclonal immunoglobulin protein, which makes them highly dependent on the ER. There are two major changes in the ER of plasma cells: the up to fivefold expansion of ER volume and the activation of compensatory mechanisms such as the unfolded protein responses (UPR) to prevent the misfolded or unfolded proteins from causing cell death.^31^ ER interact extensively with mitochondria and play a critical role in mitochondria fission and mitophagy.^32^ It was demonstrated that ER provides the phagophore membrane for autophagosome formation, thus regulating the level of mitophagy.^33^ Therefore, it is possible that when there is higher demand on the ER for monoclonal immunoglobulin production, there is less phagophore membrane provided by ER and thus less mitophagy. This is consistent with the clinical observation that in MGUS patients higher levels of M protein are associated with increased risk of transformation to MM.^34^ Additionally, reduced level of oxidative phosphorylation and decreased oxidative stress have been shown to suppress mitophagy and decrease susceptibility to apoptosis.^35^ Recently, Jin et al. found that the AAA^+^‐ATPase Atad3a suppresses Pink1‐dependent mitophagy and maintains hematopoietic homeostasis.^36^

Our data reveal that the effects of PINK1‐dependent mitophagy in MM pathogenesis are likely through the activation of the MOB1B‐mediated Hippo‐YAP/TAZ pathway. The Hippo pathway has been identified as a key regulator of cell proliferation, apoptosis, and stemness and thus cancer development.^37^ The family of MOB co‐activator proteins (MOB1a and MOB1b) is involved in mitotic exit and cell morphogenesis.^38^ Acting as a tumor suppressor and the core component of Hippo signaling, loss of MOB1 would increase cell proliferation and reduce apoptosis.^18^ MOB1B is crucial for the autophosphorylation and activation of LATS/NDR (nuclear Dbf2‐related) kinases, which phosphorylate YAP/TAZ and promote their degradation.^39^ The activity of YAP and TAZ has recently been linked to the nuclear transduction of cytoskeletal signals and has been characterized as components of the Hippo pathway.^40^ We have shown that the level of MOB1B expression increased dramatically and the expression of YAP/TAZ decreased significantly with our three mitophagy induction approaches. Using MOB1B‐specific shRNA knockdown and YAP/TAZ genetic overexpression, we have further demonstrated an important role of MOB1B‐YAP/TAZ pathway in PINK1‐mitophagy‐mediated myeloma cell migration.

It remains to be determined how PINK1‐dependent mitophagy upregulates the MOB1B Hippo‐YAP/TAZ pathway. There are several likely mechanisms. For example, the Hippo pathway can be activated by many stimuli including cell overgrowth, tension, or ROS stress. It has also been demonstrated that G‐protein‐coupled receptor engagement by lysophosphatidic acid and sphingosine 1‐phosphophate regulates the Hippo‐YAP pathway.^41^ It is therefore likely that the metabolic stress induced by the suppressed mitophagy upregulates the MOB1B Hippo‐YAP/TAZ pathway. It is also possible that the epigenetic changes caused by mitochondrial dysfunction could affect MOB1B Hippo‐YAP/TAZ pathway. Recent studies have suggested that PINK1 could directly regulate pathways that are important in DNA mismatch repair, drug resistance, and tumor progression.^42^

Several other studies have provided insights on the mechanisms of how the Hippo‐YAP/TAZ pathway regulates metastasis in solid tumors.^43^ Overexpression of YAP in breast cancer cell lines induced the expression of fibronectin, vimentin, and N‐cadherin, which are known to be important for cell adhesion and metastasis.^44^ It has also been shown that YAP upregulates the cytokines IL6, IL8, and C‐X‐C motif ligand 1, 2, and 3, promoting vascular invasion by cancer cells.^45^ Recently, Sakabe et al. found that cytoplasmic YAP positively regulated the activity of the small GTPase CDC42, and deletion of CDC42 caused severe defects in endothelial migration.^46^ YAP has been shown to promote migration through activation of Rho GTPase activating protein 29 (ARHGAP29) leading to suppression of the RhoA‐LIMK‐cofilin pathway, and by destabilizing F‐actin resulting in cytoskeletal rearrangement through alteration of the dynamics of F‐actin/G‐actin turnover.^47^ In addition, the YAP/TEAD/p65 axis,^48^ Tenascin‐C/integrin α9β1/YAP,^49^ YAP/JNK‐Drp1‐Mitochondrial Fission‐HtrA2/Omi Pathways,^50^ and CDK5/YAP^51^ were also reported to be closely linked to migration and metastases. Thus, YAP and TAZ have both emerged as important contributors in cancer metastasis and are an attractive target for treatment.^52^

Consistent with recent findings that deletion of *pink1* or *park2* promoted mutant Kras‐driven pancreatic tumorigenesis,^15^ we found that *XBP‐1s* ^+/+^/*pink1^−/−^* mice had accelerated development of myeloma manifested by increased osteoclasts, higher levels of bone marrow and spleen CD38^+^ plasma cells and serum IgG, the development of M protein and bone lytic lesions. Interestingly, when VK*MYC splenocytes were injected into *pink1* ^−/−^ knockout mice, the *pink1* ^−/−^ recipient mice had much faster development of myeloma compared to *pink1* ^+/+^ recipient mice. These data suggest that PINK1‐dependent mitophagy not only directly regulates myeloma cell migration and homing, but also affects the microenvironment that promotes myeloma tumorigenesis. Accumulating lines of evidence have demonstrated an important role of autophagy and mitophagy in T lymphocyte proliferation, the regulation of Treg cells, antigen presentation, and inflammation.^53^ It is very likely that *pink1* ^−/−^ recipient mice have defects in antitumor immunity, thus promoting myeloma development in our mouse models.

Our study demonstrates that PINK1‐dependent mitophagy can be targeted for the treatment of myeloma. Salinomycin an antibacterial and coccidiostat which induces mitophagy has been reported to have activity against solid tumors in several case reports.^17^ Our data shows that salinomycin induces mitophagy in in vitro and in vivo models of myeloma, and had activity as a single agent in a murine myeloma xenograft mouse model. Our current finding was consistent with our previous report of targeting mitophagy for overcoming bortezomib resistance in multiple myeloma.^54^ We are currently in the process of determining the efficacy of various combinations of salinomycin with other antimyeloma agents to see if these combinations lead to increased efficacy and decreased resistance.

Our study also suggests that measuring cell‐free PINK1 could serve as a surrogate biomarker of MM treatment response. We found that the median cfPINK1 DNA level increased, nearly, 2‐ to 29‐folds with CCCP or salinomycin treatment (Figure S7, Supporting Information). Of interest, as shown in Figure 9G,H, the significant association between serum cell‐free PINK1 DNA and the in vivo antimyeloma activity of salinomycin in mice suggests a potential utility of cfPINK1 DNA for therapeutic monitoring. Additional studies to explore the value of cfPINK1 as biomarker for MGUS to myeloma transformation are warranted.

In summary, we have unequivocally shown that PINK1‐dependent mitophagy plays a critical role in regulating myeloma cell migration, spreading, and tumorigenesis. We have demonstrated proof of principle of targeting mitophagy for MM treatment and utilizing cell‐free PINK1 DNA as a surrogate biomarker of mitophagy and treatment response. Our study has important implications in our understanding of myeloma pathogenesis and in our developing novel therapeutic agents for MM treatment.

## 4 Experimental Section

##### Reagents and Cell Lines

Human PINK1 short hairpin RNA (shPINK1), MAP1LC3B shRNA (shLC3B), MOB1B shRNA, YAP1 overexpressing plasmid, TAZ overexpressing plasmid, and LentiORF PINK1 overexpressing plasmid were purchased from GE Healthcare Life Science (Piscataway, NJ, USA). Control shRNA, LentiORF empty vector, pEGFP‐LC3B, packaging psPAX_2_, and envelope VSVG vector were obtained from Addgene (Cambridge, MA). Lipofectamine 2000 was purchased from Invitrogen, Carlsbad, CA. MM.1S (ATCC CRL‐2974) and MM.1R (ATCC CRL‐2975) cells were purchased from ATCC (Rockville, MD, USA). RPMI 8226 (631‐CCL‐155), NCI‐H929 (540‐CRL‐9068), and U266 (TIB‐196) were purchased from Duke Cell Culture Facility (CCF). OPM1 and RPMI 8226/Dox were gifts of Dr. Bei Liu at the Medical University of South Carolina (Charleston, SC). Anti‐PINK1 antibody (catalogue#: ab75487 and ab216144) was purchased from Abcam (Cambridge, MA); anti‐PARK2 antibody (catalogue#: 14060‐1‐AP) was purchased from Proteintech Group (Chicago, IL, USA); anti‐LC3B (catalogue#: 2775S) and anti‐YAP (catalogue#: 4912) antibodies were purchased from Cell Signaling Technology (Danvers, MA); anti‐MOB1B (catalogue#: SAB4301038) and anti‐TAZ (catalogue#: HPA007415) antibodies were purchased from Sigma‐Aldrich (St Louis, MO, USA). Carbonylcyanide‐*m*‐chlorophenylhydrazone (CCCP, catalogue#: C2759) and salinomycin (catalogue#: S4526) were purchased from Sigma Chemical Co. (St. Louis, MO). MM cell lines were routinely cultured in RPMI‐1640 medium supplemented with 10% FBS, 1% v/v penicillin, and 100 µg mL^−1^ streptomycin under ATCC recommended conditions. The HEK293 cells were maintained in Dulbecco's Modified Eagle Medium (DMEM) supplemented with 10% FBS and a 1:100 antibiotic‐antimycotic.

##### Boyden Chamber Transwell Migration Assay

Migration of MM cells was determined using 24‐well Boyden chambers (Corning) with transwell inserts of 8 × 10^−6^ m pore size as previously reported. Briefly, MM cells (40 000 cells per well) receiving the indicated treatments were seeded on the inserts with RPMI‐1640 medium containing 5% of FBS and cultured in the lower chambers with complete RPMI‐1640 culture medium at 37 °C for 4 days. The migrated cells were collected for cell counting or resuspended in 100 µL of sterile PBS solution and counted using microscope or measured at OD_490_ after 10 µL of MTT was added.

##### OCRs Measurement in Cells

The extracellular oxygen consumption rate was determined using the Seahorse XF24 extracellular flux analyzer (Agilent Seahorse Bioscience). Briefly, MM.1S cells were seeded into XF24 cell culture microplate at a density of 25 000 cells per well and incubated at 37 °C in regular media before the assay. Cell metabolic rates were measured following the sequential addition of 1 × 10^−6^ m oligomycin (Sigma 75351), 4 × 10^−6^ m carbonyl cyanide 4‐(trifluoromethoxy)‐phenylhydrazone (FCCP; Sigma, C2920), and 0.5 × 10^−6^ m rotenone (Sigma, R8875) + 0.5 × 10^−6^ m antimycin A (Sigma, A8674). For the OCRs and ECARs, the baseline mitochondrial respiration was established by recording extracellular oxygen concentration at several time‐points and normalized to protein concentration using the Protein Assay reagent (Bio‐Rad, 500‐0006) and expressed as % from baseline. Proton leak, ATP production, and spare respiratory capacity were also evaluated using the Seahorse XF according to the manufacturer's instructions.

##### Mice

Breeding pair of pink1 knockout mice (*pink1^−/−^*, RRID: IMSR_JAX: 017946) and C57BL/6 WT controls were purchased from the Jackson Laboratory (Bar Harbor, Maine). Eµ‐XBP‐1s transgenic mice (*XBP‐1s^+/+^*) on a C57BL/6J background were kindly provided by Dr. Bei Liu at the Medical University of South Carolina. NSG at 8–10 weeks of ages were obtained from the Jackson Laboratory. Mice were fed standard chow ad libitum and kept on a 12 h light, 12 h dark cycle. All protocols for mouse experiments were reviewed and approved under Animal Protocol Number A097‐17‐04 by the Institutional Animal Care and Use Committee of Duke University Medical Center.

##### Myeloma Xenograft Mouse Model

MM.1S myeloma cells stably expressing luciferase (5 × 10^5^) were injected via tail vein into the NSG mice that had previously received 1.5 Gy total body irradiation from a ^137^Cs source. Tumor development was measured and quantified every 3 days by IVIS Lumina XR with a Caliper Xenogen Spectrum instrument at Duke University Optical Molecular Imaging and Analysis (OMIA) Shared Resource Center.

##### XBP‐1s^+/+^pink1^−/−^ Mice and XBP‐1s^+/+^pink1^+/+^ Mice

Eµ‐XBP‐1s transgenic mice and *pink1^−/−^* mice were crossbred at the animal facility and the offspring genotyped. *XBP‐1s^+/+^pink1^−/−^* mice and *XBP‐1s^+/+^pink1^+/+^* mice were monitored every week and sacrificed at 10 months old.

##### Syngeneic Transplantable VK*MYC Myeloma Mouse Model

Cryopreserved splenocytes of VK*MYC mice were kindly provided by Dr. P. Leif Bergsagel at Mayo Clinic (Arizona). Cells were expanded on C57BL/6 mice. VK*MYC splenocytes were injected via tail vein into 10–12 weeks old of *pink1^−/−^* knockout mice and their WT littermate controls (10^6^ cells per mouse).

##### Bone Marrow Osteoclast Differentiation

Osteoclasts were differentiated from bone marrow cells of *XBP‐1s^+/+^pink1^−/−^* and *XBP‐1s^+/+^pink1^+/+^* mice (8–12 weeks old). Cells were differentiated with 40 ng mL^−1^ of mouse macrophage colony‐stimulating factor (M‐CSF, R&D Systems) in α‐MEM containing 10% FBS for 3 days, and additional 100 ng mL^−1^ of mouse RANKL (Sigma‐Aldrich) was added to continue culture for additional 3 days. Tartrate‐resistant acid phosphatase (TRAP) staining of osteoclasts was performed using a leukocyte acid phosphatase staining kit (Sigma‐Aldrich).

##### Bone Imaging with X‐Ray and Micro‐CT

High‐resolution X‐ray computed tomography scanning was performed to evaluate bone lytic lesions using a Nikon's XT H 225 ST scanner as previously described.^55^ Briefly, mouse tibiae were removed and scanned using both X‐ray imaging (2D) in real time and µCT scanning at a resolution of 7 µm. Trabecular bone density was calculated using Avizo software (Visualization ScienceGroup, Burlington, MA).

##### RNA Isolation and Gene Expression Analyses

Total RNA was isolated from cells using RNeasy mini kit (Qiagen, Hilden, Germany) according to the manufacturer's protocol. RNA (1000 ng) was reverse‐transcribed into cDNA using a cDNA synthesis kit (iScript cDNA Synthesis kit, Bio‐Rad). Gene expression analysis was then performed on the QuantStudio 6 Flex Real‐Time PCR System (Applied Biosystems). Each reaction was performed in triplicate in a 384‐well plate (Deville Scientific, Metuchen, NJ). The expression of each gene was normalized to the expression of 18S rRNA.

##### Serum Protein Electrophoresis and ELISA

Mouse serum samples were collected from *XBP‐1s^+/+^pink1^−/−^* and *XBP‐1s^+/+^pink1^+/+^* mice. M protein was measured by protein electrophoresis according to the manufacturer's instruction. Serum IgG level was measured by ELISA kit according to the manufacturer's protocol.

##### qPCR Array

MM.1S cells were transfected with LentiORF PINK1 plasmid containing w/ Stop Codon (CloneId:PLOHS_100003663) and LentiORF empty vector, and incubated with 10 µg mL^−1^ Blasticidin S (Thermo Fisher Scientific, Waltham, MA) for 2 weeks. Total mRNA was isolated and reverse‐transcribed into cDNA. Gene expression was conducted by 96‐well qPCR arrays for genes involved in human adhesion and Hippo signaling pathways RT2 Profiler PCR Array (Qiagen, Hilden, Germany). Differently expressed genes were screened out by RT2 Profiler PCR Array Data Analysis web portal (https://www.sabiosciences.com/pcr/arrayanalysis.php).

##### Confocal Microscopy Analysis

MM cells (2 × 10^3^) transduced with LC3B‐EGFP vector were placed on slides by Cytospin (Shandon Southern Instruments, Runcorn, Cheshire, UK) and fixed with 4% paraformaldehyde in PBS for 15 min. Cells were then washed with PBS, permeabilized with 0.2% Triton X‐100 in PBS for 5–10 min, blocked with 5% BSA in PBS for 30 min, followed by incubation with MitoTracker Deep Red FM 633 (catalogue#: M22426, Invitrogen) in the dark at 37 °C. Coverslips were PBS‐washed and mounted on microscope slides using Permount without DAPI (Fisher Scientific). For data acquisition, images were obtained from Leica STED and confocal system (Leica Microsystems, Wetzlar, Germany). Image analysis was performed using Leica Application Suite Advanced Fluorescence (LAS AF) software. For LC3B and mitochondria puncta counting, perinuclear and peripheral cell boundaries were manually traced.

##### Intravital Two‐Photon Confocal Microscopy Imaging

MM.1S myeloma cells (5 × 10^6^) were incubated with DiR lipophilic dye (Invitrogen) according to previous publications.[qv: 16e] At the end of the incubation, cells were washed twice in warm PBS, counted, and resuspended in PBS at a concentration of 5 × 10^6^ mL^−1^ for engraftment. Dye labeling efficiency was tested by flow cytometry.

For the two‐photon intravital imaging, DiR‐labeled MM.1S cells (5 × 10^5^) were injected into NSG mice via tail vein.[qv: 16e] Mice were anesthetized and a rectangular incision was made in the scalp. To label the blood vessel, dextran (Dex‐FITC) was administered by tail vein injection. Mice were placed in a specially designed restrictor with a heating pad, and a coverslip was placed over the exposed calvarial bone. Images were obtained through the intact mouse skull using a Leica SP5 confocal and multiphoton microscope with a 20×/0.40 NA (numerical aperture) objective lens. The system uses a femtosecond titanium:sapphire laser (Chameleon) for multiphoton or single‐photon excitation and multiple Cs lasers (including an argon laser, a HeNe laser, and 561 and 633 nm diode lasers) for single‐photon excitation. Images were captured with Leica LAS‐AF software using line and frame averaging. The calvarial BM was subdivided into numbered anatomical areas, and overlapping 20× images were captured of the entire region. After the procedure, these images were merged to generate a montage image of the entire calvarium, and cell homing and mobilization counts were obtained.

##### Western Blot Analysis

Cells under different treatments were lysed in RIPA buffer (Sigma‐Aldrich, St Louis, MO, USA) for protein extraction, and protein concentration was determined by the DC Protein Assay (Bio‐Rad). 20 µg of protein samples were separated on 12% SDS polyacrylamide gels, and the transferred to PVDF membranes and incubated with PINK1 (1:200), PARK2 (1:500), LC3B (1:1000), MOB1B (1:1000), YAP (1:1000), TAZ (1:500), β‐actin (1:10 000), and GAPDH (1:10 000) overnight at 4 °C. Membranes were then washed three times using TBST and incubated with anti‐HRP‐conjugated secondary antibodies (1:10 000) for 2 h at room temperature before signal detection by chemiluminescent substrate (catalogue#: TD263834 and SA241929, Thermo Scientific).

##### Mitochondrial Membrane Potential (Δψm) Analysis

Mitochondrial membrane potential (Δψm) was determined by JC‐1 fluorescent probe kit (Molecular Probes, Eugene, OR, USA) as previously described.^54^ Cells receiving different treatments were incubated with 2 × 10^−6^ m of JC‐1 dye in warm RPMI1640 media at 37 °C for 15 min. Cell pellets were resuspended in cold PBS and read on a BD FACS Canto II flow cytometer (BD Biosciences) using 529 and 590 nm emission peaks for monomeric and JC‐aggregate forms, respectively.

##### TEM Analysis

TEM analysis was performed to monitor the mitochondria network. MM cells receiving different treatments were resuspended and washed using HBSS buffer three times at room temperature and fixed with TEM fixative (10 mL 20% formaldehyde, 4 mL 25% glutaraldehyde, 5 mL 10 × PBS, 0.01% malachite green, and 31 mL distilled water) for at least 2 h at room temperature or overnight at 37 °C. The fixative was removed and samples were washed with PBS, postfixed by OSO_4_, stained by uranyl acetate, dehydrated by alcohol, and embedded by araldite. Thin sections of samples were visualized using a FEI Tecnai G² Twin electron microscope (FEI, Hillsboro, Oregon).

##### Cell‐Free DNA Extraction and Cell‐Free PINK1 Expression Analysis

Cell‐free DNAs from cell culture medium and mouse plasma samples were isolated using Qiagen circulating nucleic acid kits (Qiagen, Hilden, Germany) according to the user manual. For cell‐free PINK1 expression analysis, QuantStudio 6 Flex Real‐Time PCR System (Applied Biosystems) was used based on 30 ng of total cell‐free DNA. Each reaction was performed in triplicate in a 384‐well format. The expression of each gene was normalized to the expression of 18S rRNA.

##### Oncomining Microarray Analysis

Clinical data of human MM were downloaded from Oncomine (https://www.oncomine.org/) and Gene Expression Omnibus (GEO, https://www.ncbi.nlm.nih.gov/geo/). For expression analysis, expression of *PINK1* and *PARK2* were analyzed in MGUS or MM patients CD138^+^ plasma cells. For the survival analysis, the overall survival time was defined as the time from diagnosis to MM specific death.

##### Statistical Methods

Each experiment was performed at least three times and represented as mean ± standard error of the mean (SEM) using Student's *t*‐test. For in vivo experiments with ≥3 groups, statistical analyses were performed with ANOVA followed by the post hoc Tukey pairwise comparisons. The *p* values were designated exactly as shown in the figures as follows: *p < 0.05; **p < 0.01; ***p < 0.005; ****p < 0.001; n.s. not statistically significant (*p* ≥ 0.05).

## Conflict of Interest

Y.K. received research funding from InCyte Corporation and Consultancy fee from Takeda Oncology USA. All other authors declare no competing conflicts of interest.

## Author Contributions

S.F. and Y.K. initiated the research, developed the concept of the paper, designed the study, and analyzed and interpreted the data; T.P. performed the intravital two‐photon confocal microscopy imaging; W.H. performed the mice irradiation and part of the flow cytometry experiment; M.P. performed TEM sample preparation and imaging; J.W. performed the seahouse XF; J.W., P.S., B.P., and D.F. assisted with experiments; S.F., N.M., N.C., D.S., and Y.K. were involved in experimental design and data interpretation; S.F. and Y.K. wrote the manuscript. All the authors approved the final manuscript.

## Supporting information

Supporting Information

Supplemental Video 11

Supplemental Video 12

Supplemental Video 13
