# Supplementary material for: PINK1‐Dependent Mitophagy Regulates the Migration and Homing of Multiple Myeloma Cells via the MOB1B‐Mediated Hippo‐YAP/TAZ Pathway
Source: Adv Sci (Weinh). 2020 Jan 23;7(5):1900860. doi: 10.1002/advs.201900860 (PMC7055555; doi:10.1002/advs.201900860)
Supplement: Supplementary file 1 — Supporting Information [file ADVS-7-1900860-s001.pdf]

Copyright WILEY-VCH Verlag GmbH & Co. KGaA, 69469 Weinheim, Germany, 2020.

ADVANCED SCIENCE
Open Access

Supporting Information

for *Adv. Sci.,* DOI: 10.1002/advs.201900860

PINK1-Dependent Mitophagy Regulates the Migration and Homing of Multiple Myeloma Cells via the MOB1B-Mediated Hippo-YAP/TAZ Pathway

*Shengjun Fan, Trevor Price, Wei Huang, Michelle Plue, Jonathan Warren, Pasupathi Sundaramoorthy, Barry Paul, Daniel Feinberg, Nancie MacIver, Nelson Chao, Dorothy Sipkins, and Yubin Kang**

PINK1-dependent mitophagy regulates the migration and homing of multiple myeloma cells *via* the MOB1B mediated Hippo-YAP/TAZ pathway

Shengjun Fan[1], Trevor Price[1], Wei Huang[1], Michelle Plue[2], Jonathan Warren[3], Pasupathi Sundaramoorthy[1], Barry Paul[1], Daniel Feinberg[1], Nancie MacIver[3], Nelson Chao[1], Dorothy Sipkins[1], and Yubin Kang[1, *]

Supplementary materials

Supplementary Legends:

Supplementary Figure 1: Level of PNK1-dependent mitophagy (i.e., *PINK1* expression) and correlation of PINK1-dependent mitophagy with overall survival in patients with MM. A, B) *PINK1* expression levels in MGUS plasma cells and myeloma cells in the Fonseca microarray datasets. Expression levels are presented as scatter plot and were compared using an unpaired Student's *t* test. C, D) Kaplan-Meyer analysis of overall survival in the Arkansas dataset basing on the expression of *PINK1* and *PARK2* in $CD138^+$ cells of myeloma patients. Survival analysis was

performed using a log-rank test. High and low *PINK1* expression (ID: 209018_s_at) was defined using a cut-off of 1071.4. High and low *PARK2* expression (ID: 1555668_a_at) was defined using a cut-off of 181.9.

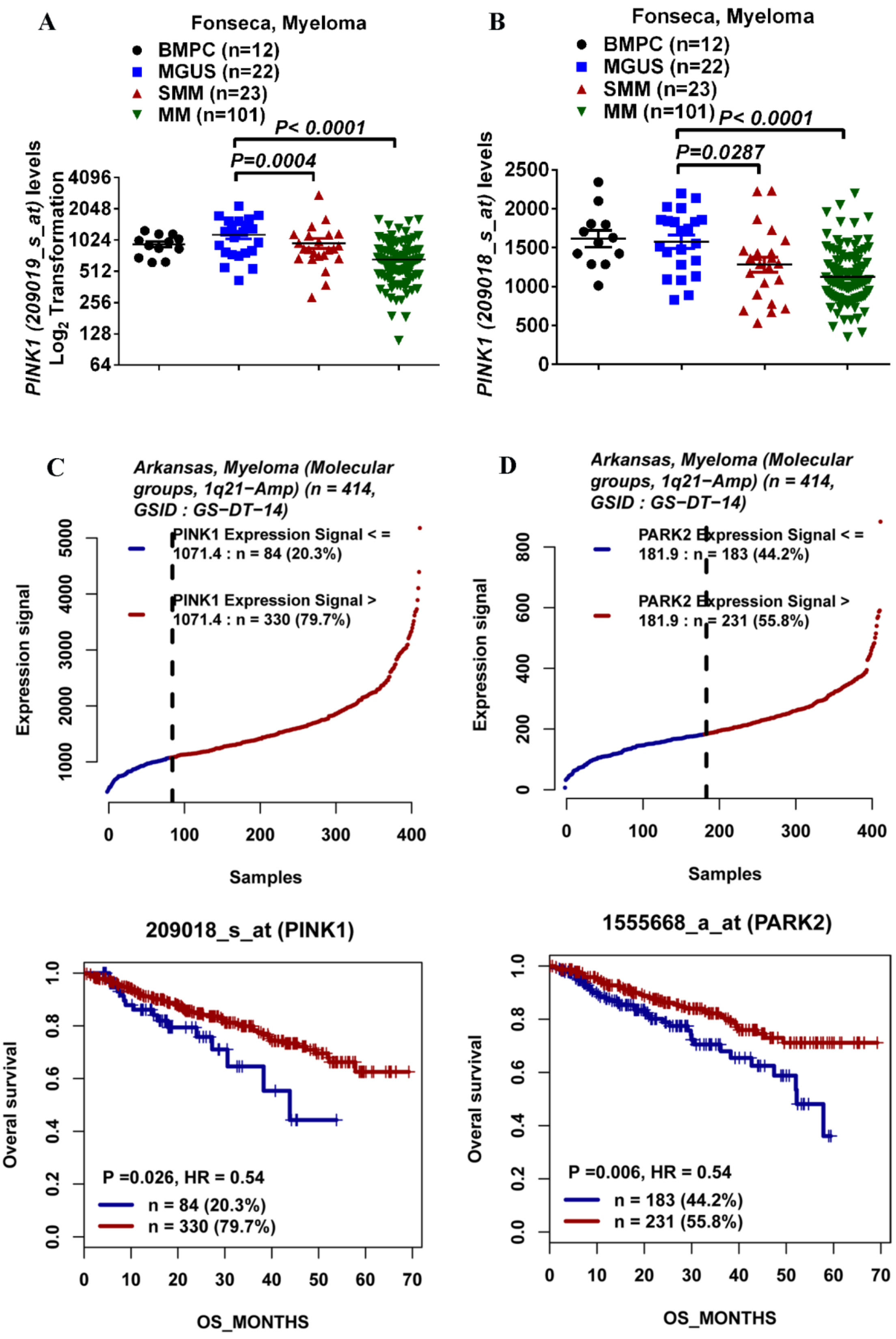
A
Fonseca, Myeloma
BMPC (n=12)
MGUS (n=22)
SMM (n=23)
MM (n=101)
P< 0.0001
P=0.0004
PINK1 (209019_s_at) levels
Log2 Transformation
B
Fonseca, Myeloma
BMPC (n=12)
MGUS (n=22)
SMM (n=23)
MM (n=101)
P< 0.0001
P=0.0287
PINK1 (209018_s_at) levels
C
Arkansas, Myeloma (Molecular groups, 1q21−Amp) (n = 414, GSID : GS−DT−14)
PINK1 Expression Signal < = 1071.4 : n = 84 (20.3%)
PINK1 Expression Signal > 1071.4 : n = 330 (79.7%)
Expression signal
Samples
D
Arkansas, Myeloma (Molecular groups, 1q21−Amp) (n = 414, GSID : GS−DT−14)
PARK2 Expression Signal < = 181.9 : n = 183 (44.2%)
PARK2 Expression Signal > 181.9 : n = 231 (55.8%)
Expression signal
Samples
209018_s_at (PINK1)
Overal survival
P =0.026, HR = 0.54
n = 84 (20.3%)
n = 330 (79.7%)
OS_MONTHS
1555668_a_at (PARK2)
Overal survival
P =0.006, HR = 0.54
n = 183 (44.2%)
n = 231 (55.8%)
OS_MONTHS

Figure S1

Supplementary Figure 2. CCCP and salinomycin induced mitophagy in myeloma cell lines. A) Mitochondrial membrane depolarization measured by MitoProbe JC-1 dye. MM cells were treated with either 5 μM CCCP or 2.5 μM salinomycin for 48 h. One of 3 representative experiments was shown. Upper right quadrant represented cells with normal mitochondrial potential and right lower quadrant represented cells having depolarized mitochondria. B, C) mRNA and protein levels of PINK1 and PARK2 expression levels in MM cells treated with either 5 μM CCCP or 2.5 μM salinomycin for 48 h. Upper panel: mRNA levels of PINK1 and PARK2 in MM.1S treated with CCCP or salinomycin (B). Lower panel: protein levels of PINK1, PARK2, LC3B-I, and LC3B-II in MM cell lines treated with either 5 μM CCCP (left) or 2.5 μM salinomycin (right) for 48 h. D) CCCP or salinomycin treatment inhibited OCR and ECAR and reduced proton leak, ATP production and spare respiratory capacity in MM cells. MM.1S cells were treated with various concentrations of CCCP (left) or salinomycin (right) for 48 h and mitochondrial respiration/mass was measured by seahorse assay. Data represented mean ± SD, n=3. *$P < 0.05$, **$P < 0.01$ and *** $P < 0.001$ compared with the DMSO control. E) Confocal microscope examination of the fusion of mitochondria and lysosomes in MM.1S cell treated with DMSO, CCCP or salinomycin. MM cell lines were transduced with MitoTracker (deep red color to label mitochondria) and LC3B-eGFP

(green color) constructs. The cells were then treated with DMSO, CCCP (5µM) or Sal (2.5µM) for 48 h and mitophagy (i.e., the fusion of mitochondria and lysosome, represented by the merged yellow/orange color) was detected by confocal microscope. F) TEM examination of the fusion of mitochondria and lysosome. MM cell lines were treated with DMSO, CCCP (5µM) or Sal (2.5µM) for 48 h and mitophagy (i.e., the fusion of mitochondria, represented as red asterisks, with lysosome, represented by yellow arrows) was detected by TEM.

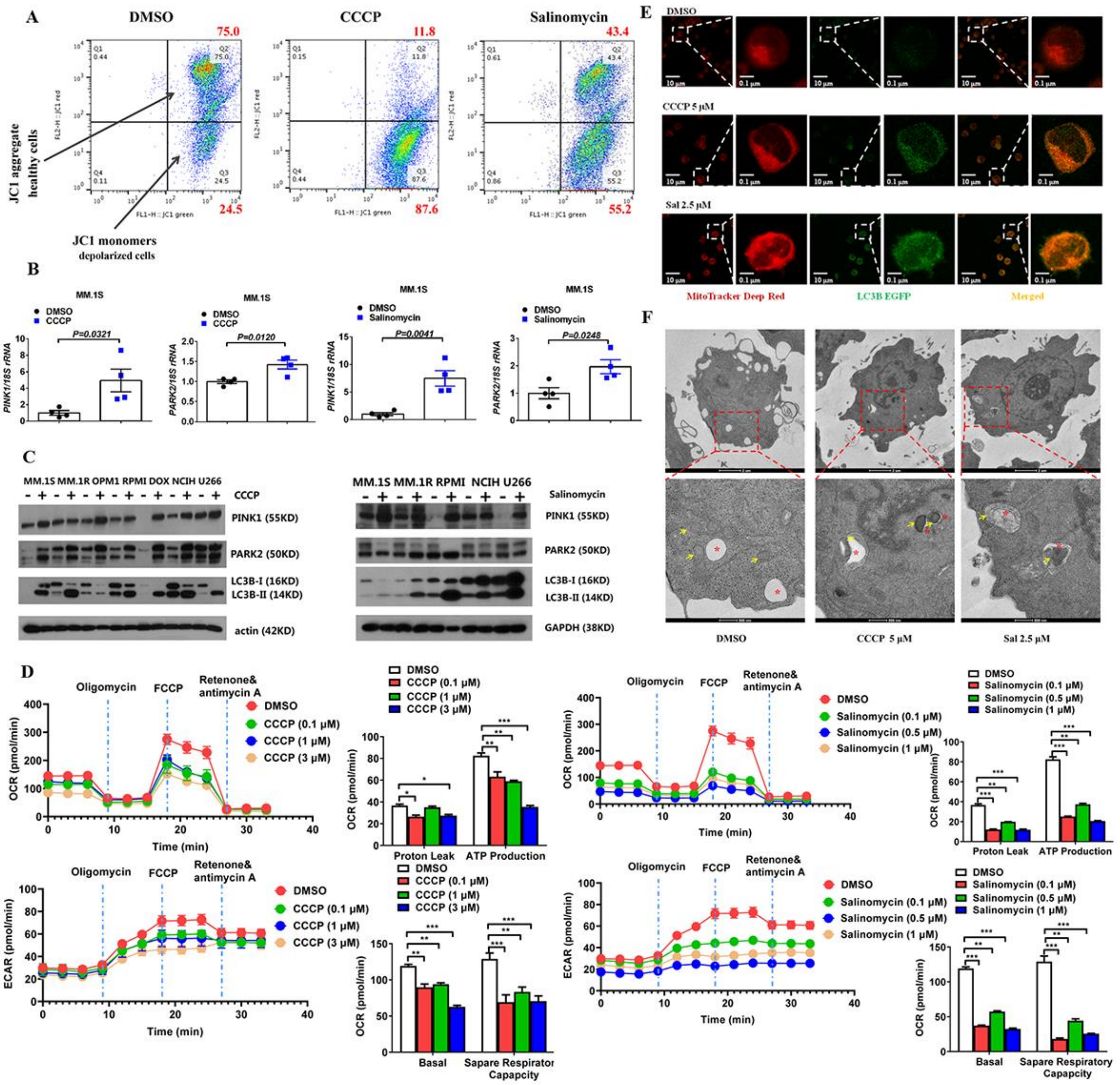
A
DMSO
CCCP
Salinomycin
75.0
24.5
11.8
87.6
43.4
55.2
JC1 aggregate healthy cells
JC1 monomers depolarized cells
B
MM.1S
DMSO
CCCP
Salinomycin
P=0.0321
P=0.0120
P=0.0041
P=0.0248
PINK1/18S rRNA
PARK2/18S rRNA
C
MM.1S MM.1R OPM1 RPMI DOX NCIH U266
CCCP
MM.1S MM.1R RPMI NCIH U266
Salinomycin
PINK1 (55KD)
PARK2 (50KD)
LC3B-I (16KD)
LC3B-II (14KD)
actin (42KD)
GAPDH (38KD)
D
Oligomycin
FCCP
Retenone& antimycin A
OCR (pmol/min)
ECAR (pmol/min)
Time (min)
DMSO
CCCP (0.1 μM)
CCCP (1 μM)
CCCP (3 μM)
Salinomycin (0.1 μM)
Salinomycin (0.5 μM)
Salinomycin (1 μM)
Proton Leak
ATP Production
Basal
Sapare Respiratory Capapcity
E
DMSO
CCCP 5 μM
Sal 2.5 μM
MitoTracker Deep Red
LC3B EGFP
Merged
F
DMSO
CCCP 5 μM
Sal 2.5 μM

Figure S2

Supplementary Figure 3. Cell proliferation, cell cycle and apoptotic analyses in MM cells under genetic forced expression of PINK1 or PARK2. A) Genetic forced expression of PINK1 has no significant effects on MM cell proliferation. Cell proliferation was monitored using MTT assay for up to 5 days on MM.1S, MM.1R, NCIH929 and U266 cells. B) Genetic forced expression of PARK2 has no significant effects towards MM cell proliferation. Cell proliferation was monitored using MTT assay for up to 5 days on MM.1S, MM.1R, NCIH929 and U266 cells. C) Genetic forced expression of PINK1 has minimal effects on cell cycle. For cell cycle, propidium iodide (PI) DNA staining was measured using flow cytometric analysis with an emission of 605 nm. D) Genetic forced expression of PINK1 has no significant effects towards MM cell apoptosis. Cell apoptosis was performed using PI and Annexin V on MM.1S, MM.1R, NCIH929 and U266 cells.

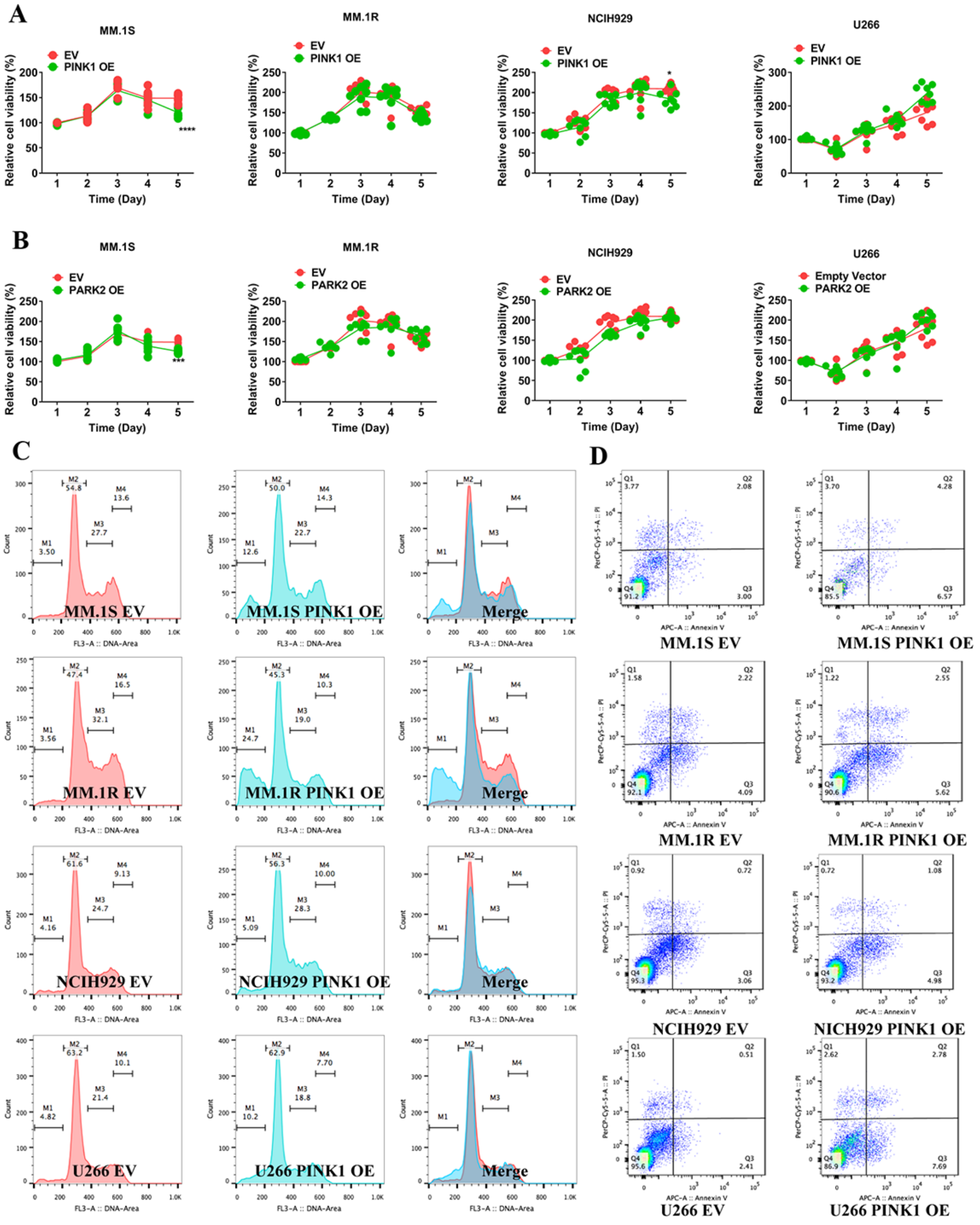
A
B
C
D
MM.1S
MM.1R
NCIH929
U266
EV
PINK1 OE
PARK2 OE
Empty Vector
Relative cell viability (%)
Time (Day)
MM.1S EV
MM.1S PINK1 OE
MM.1R EV
MM.1R PINK1 OE
NCIH929 EV
NCIH929 PINK1 OE
U266 EV
U266 PINK1 OE
Merge
NICH929 PINK1 OE

Figure S3

Supplementary Figure 4. PINK1-mediated mitophagy induction inhibited myeloma cell migration. MM.1S, MM.1R, NCIH929 and U266 were transduced with control vector, PINK1 OE vector, PINK1 stably OE with shPINK1, or PINK1 stably OE with shLC3B. Cell transwell migration assay was performed. Y-axis represented migrated cells in the lower chamber by MTT absorbance (A, left panel) or cell counts (B, right panel). Data represent mean ± SEM, n=3.

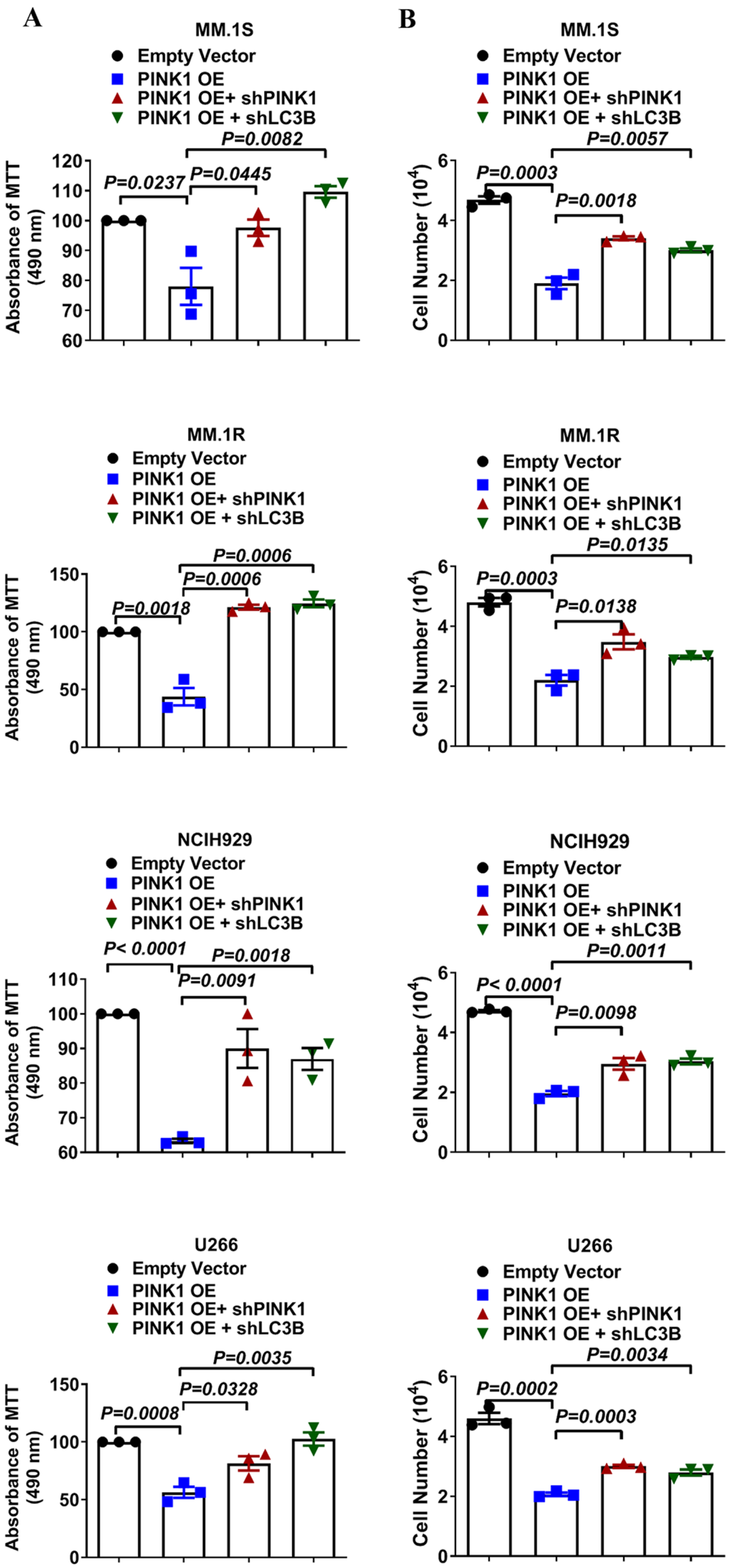
A
B
MM.1S
Empty Vector
PINK1 OE
PINK1 OE+ shPINK1
PINK1 OE + shLC3B
P=0.0237
P=0.0445
P=0.0082
Absorbance of MTT (490 nm)
P=0.0003
P=0.0018
P=0.0057
Cell Number (10^4)
MM.1R
P=0.0018
P=0.0006
P=0.0006
P=0.0003
P=0.0138
P=0.0135
NCIH929
P< 0.0001
P=0.0091
P=0.0018
P< 0.0001
P=0.0098
P=0.0011
U266
P=0.0008
P=0.0328
P=0.0035
P=0.0002
P=0.0003
P=0.0034

Figure S4

Supplementary Figure 5. Treatment with CCCP or salinomycin inhibited migration and proliferation in myeloma cell lines. A-F) CCCP or salinomycin treatment reduced MM cell *in vitro* migration. MM cells were treated with DMSO, CCCP (5μM) or Sal (2.5μM) for 48 h, and cells in the lower transwell chamber were collected and cell number was determined by MTT absorbance. G) CCCP treatment inhibited MM cell proliferation. Seven MM cells were treated with various concentrations of CCCP for 24 or 48 h, and cell survival was examined by MTT. H) Salinomycin treatment inhibited MM cell proliferation. Seven MM cells were treated with various concentrations of salinomycin for 24 or 48 h, and cell survival was examined by MTT. Data represent mean ± SEM, n=3-6. *$P < 0.05$, **$P < 0.01$ and *** $P < 0.001$ compared with the DMSO group (control).

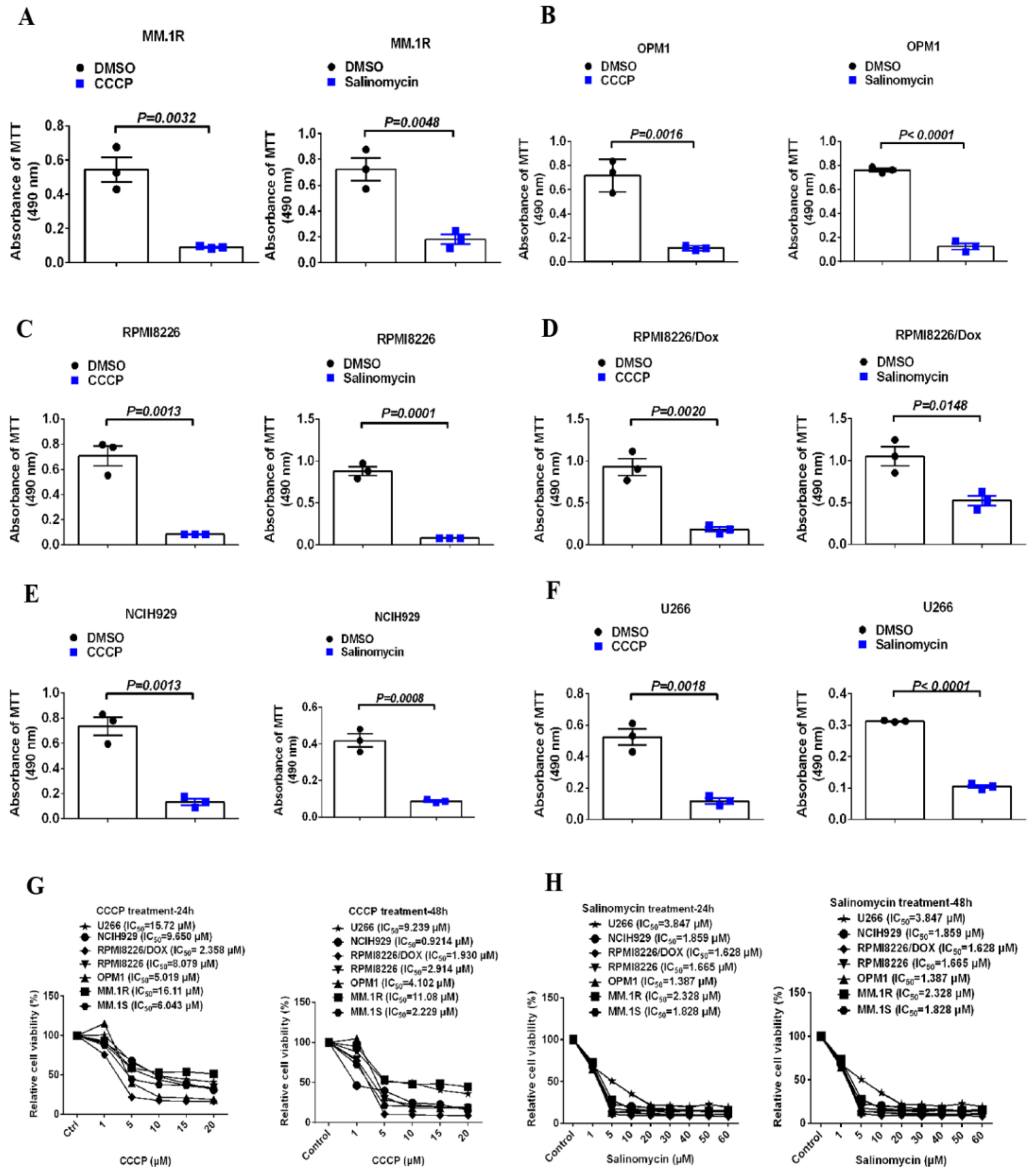

Figure S5

Supplementary Figure 6. PINK1 over-expression up-regulated MOB1B mRNA expression in MM cells. MM cells were transduced with control empty vector, PINK1 OE vector, or PINK1 OE vector + PINK1 shRNA (rescue group). Data represent mean ± SEM, n=3.

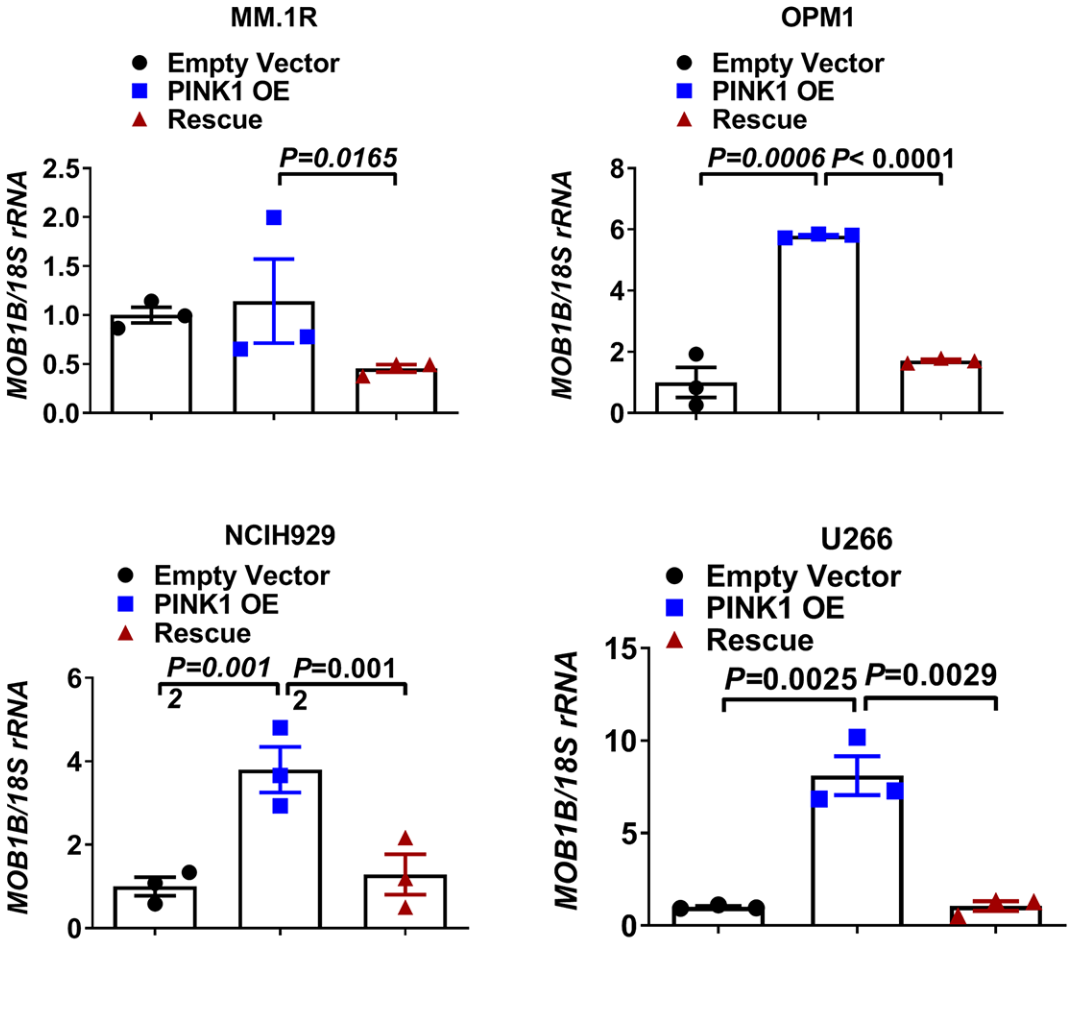

Figure S6

Supplementary Figure 7. cfPINK1 DNA as a biomarker for PINK1-dependent mitophagy. Cell free PINK1 DNA was measured in the culture medium of: A) MM.1S cells treated with CCCP. B) MM.1S cells treated with salinomycin. C) MM.1S cells transduced with control vector, PINK1 OE, or PINK1 OE+PINK1shRNA (rescue group). Data represent mean ± SEM, n=3-6.

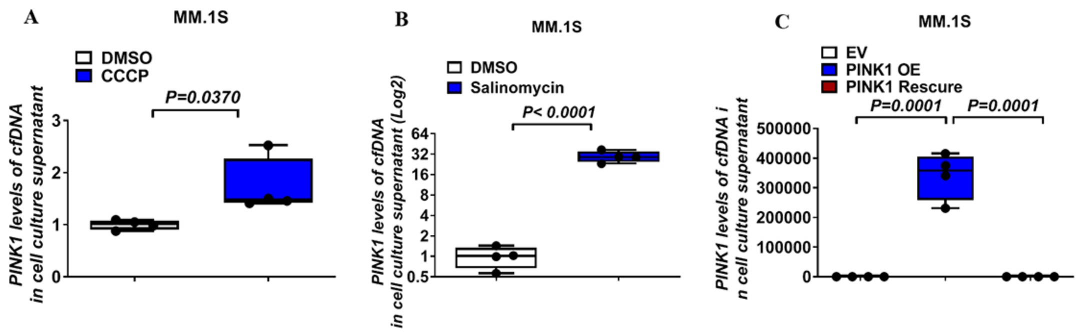

Figure S7

Supplementary Figure 8. Montage image of mouse calvarial region 2 hours after administration of MM.1S myeloma cells transduced with the empty LentiORF vector. Representative lateral regions corresponding to the images are presented in Figure 4A-a (blue dots were DiR-labeled MM.1S myeloma cells; green represented dextran-FITC vasculature, scale bar = 200 μm).

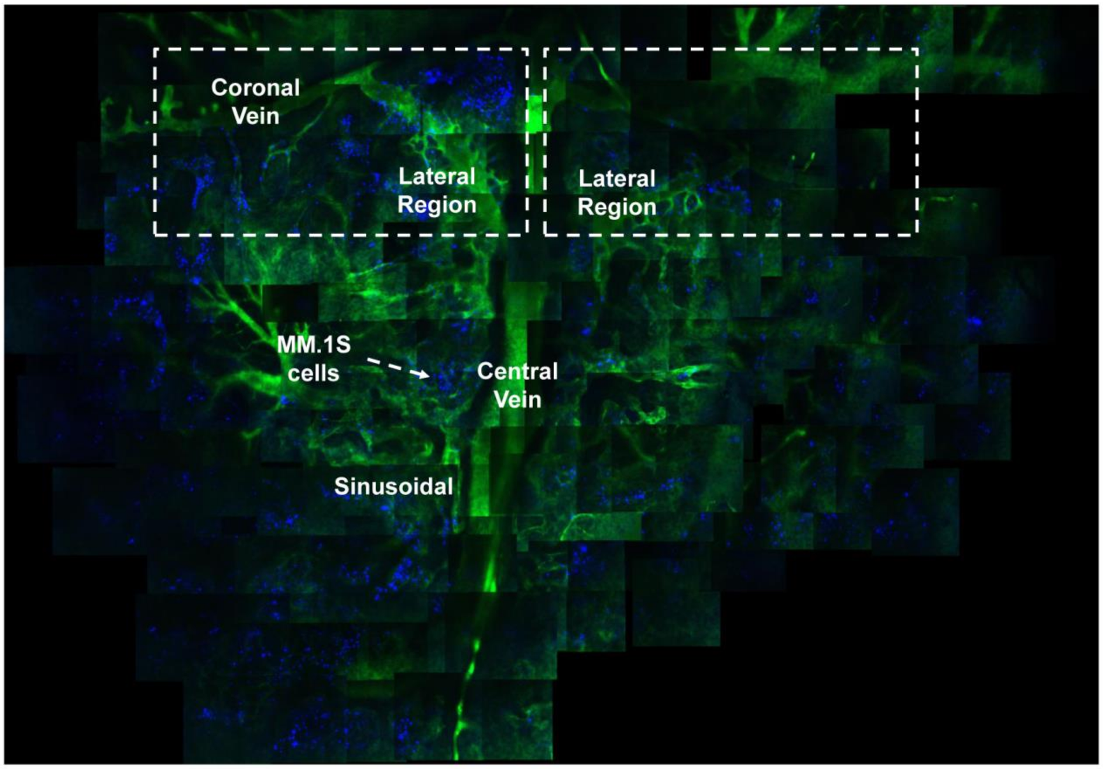

Figure S8

Supplementary Figure 9. Montage image of mouse calvarial region 2 hours after administration of MM.1S myeloma cells transduced with the PINK1 over-expressing (OE) vector. Representative lateral regions corresponding to the images are presented in Figure 4A-b (blue dots were DiR-labeled MM.1S myeloma cells; green represented dextran-FITC vasculature, scale bar = 200 μm).

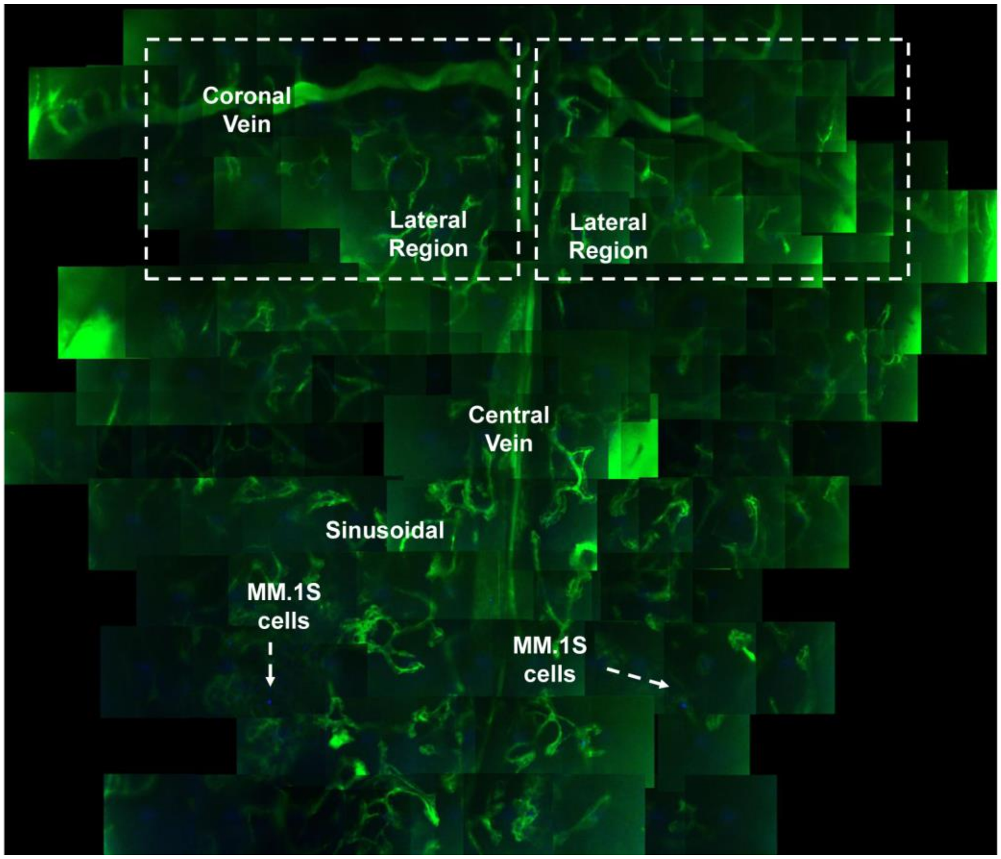

Figure S9

Supplementary Figure 10. Montage image of mouse calvarial region 2 hours after administration of MM.1S myeloma cells transduced with the PINK1 OE vector followed by shPINK1 (PINK1 rescue). Representative lateral regions corresponding to the images are presented in Figure 4A-c (blue dots were DiR-labeled MM.1S myeloma cells; green represented dextran-FITC vasculature, scale bar = 200 μm).

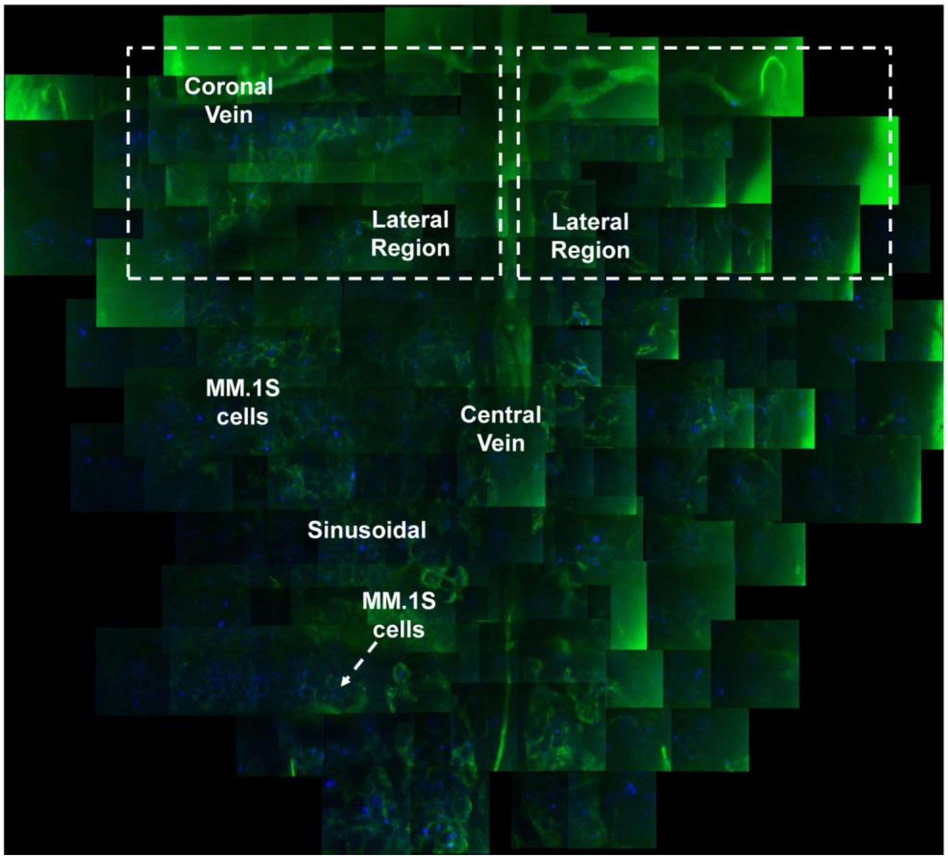

Figure S10

Supplementary Video 11. Real-time *in vivo* video of the calvarial BM of NSG mice engrafted with fluorescent MM.1S cells transfected with empty vector plasmid (2 hours post-engraftment). Intravital confocal microscopy performed through a thinned-skull window in a mouse approximately 2 h post-intravenous engraftment of MM.1S-DiR cells. Circulating MM.1S-DiR cells (blue) are visible in the central vein and in the lateral region of the calvarial system.

Supplementary Video 12. Real-time *in vivo* video of the calvarial BM of NSG mice engrafted with fluorescent MM.1S cells transfected with PINK1 over-expression plasmid (2 hours post-engraftment). Intravital confocal microscopy performed through a thinned-skull window in a mouse approximately 2 h post-intravenous engraftment of MM.1S-DiR cells. Circulating MM.1S-DiR cells (blue) are visible in the central vein, but not in the lateral region of the calvarial system.

Supplementary Video 13. Real-time *in vivo* video of the calvarial BM of NSG mice engrafted with fluorescent MM.1S cells transfected with PINK1 over-expression and shPINK1 plasmids (2 hours post-engraftment). Intravital confocal microscopy performed through a thinned-skull window in a

mouse approximately 2 h post-intravenous engraftment of MM.1S-DiR cells. Circulating MM.1S-DiR cells (blue) are visible in the central vein and in the lateral region of the calvarial system.

Supplementary Table. Human primers used in this study.

Name	Seq 5->3
h18S rRNA-F	GTAACCCGTTGAACCCCATT
h18S rRNA-R	CCATCCAATCGGTAGTAGCG
hPINK1-F	GTGGACCATCTGGTTCAACAGG
hPINK1-R	GCAGCCAAAATCTGCGATCACC
hPARKIN-F	CCAGAGGAAAGTCACCTGCGAA
hPARKIN-R	CTGAGGCTTCAAATACGGCACTG
hND1-F	CCA ACC TCC TAC TCCTCA TTG T
hND1-R	GGG AAT GCT GGAGAT TGT AAT G
hD310-F	ACA ATT GAA TGT CTGCAC AGC CAC TT
hD310-R	GGC AGA GAT GTG TTTAAG TGC TG
hAMOTL1-F	GTCTACCACCAAGCGAGAATCG
hAMOTL1-R	CTGCTGGATAGTTGCCTGTTAGC
hFAT4-F	CCCCACATTTGCCAGTAAAGCG
hFAT4-R	CGTGAACTGAGAGTTTCCACCG
hFJX1-F	GCTCGTAAGCAACCTCTTCAGC
hFJX1-R	CTTGTCCCACATGCCTGCTACC
hLATS2-F	GTTCTTCATGGAGCAGCACGTG
hLATS2-R	CTGGTAGAGGATCTTCCGCATC
hMEIS1-F	AAGCAGTTGGCACAAGACACGG
hMEIS1-R	CTGCTCGGTTGGACTGGTCTAT
hMOB1B-F	TTCGGATGGCTGTCATGCTTCC
hMOB1B-R	GCTGACATCACTGGACAACTCTC
hMPDZ-F	TTACCAGAGCGAGAAGAGGGAG
hMPDZ-R	GCTTGGTTCTCTCCAGAGTTCC
hPTPN14-F	AGTGTGGTGAGCACTACTCGGA
hPTPN14-R	CTACACACGCTGCCATTGGTGA
hRASSF2-F	CATCCGTGTTCACACCAGCCTA
hRASSF2-R	GACCACGTACAAGGCAAACTCC
hYAP-F	TGTCCCAGATGAACGTCACAGC
hYAP-R	TGGTGGCTGTTTCACTGGAGCA
hTAZ-F	ACCGTGTCCAATCACCAGTCCT
hTAZ-R	CCTTGGTGAAGCAGATGTCTGC
